# Supplementary material for: Estimating Transcriptome Diversity and Specialization in Capsicum annuum L
Source: Plants (Basel). 2024 Mar 29;13(7):983. doi: 10.3390/plants13070983 (PMC11013594; doi:10.3390/plants13070983)
Supplement: Supplementary file 1 [file plants-13-00983-s001.zip › plants-2894157-supplementary.pdf]

SUPPLEMENTARY INFORMATION FOR
"Estimating Transcriptome Diversity and Specialization in *Capsicum annuum* L."

NEFTALÍ OCHOA-ALEJO, M. HUMBERTO REYES-VALDÉS AND OCTAVIO MARTÍNEZ

In the main text of the paper, sections of this document are referred as "**Supplementary SI.#**". The Appendix in SI.5 presents boxes with R code to replicate all results. All objects produced as results of the R computations are available upon request in the binary R object "`ObjectsForSupplementary.RData`".

Content

SI.1.	Sample size sensitivity analysis	1
SI.2.	Global analysis per library	3
SI.3.	Analyses by genotype	4
SI.3.1.	Diversity and Specialization	4
SI.3.2.	Study of locus specificity among sources	6
SI.3.3.	Gene specificity by type	10
SI.4.	Analyses by time of development	12
SI.4.1.	Highly specific genes per time of fruit development	14
SI.5.	Appendix: Boxes with R code	19
SI.5.1.	Computations for "Sample size sensitivity analysis"	19
SI.5.2.	Computations for "Global analysis per library"	22
SI.5.3.	Computations for "Analyses by genotype"	25
SI.5.4.	Computations for "Analyses by time of development"	38

SI.1. Sample size sensitivity analysis

In RNA-Seq sample size is also known as "*sequencing depth*", and it refers to the number of clean reads mapped to the reference genome and employed to estimate relative gene expression in each library. The sample size of each RNA-Seq library depends on many factors, among the most important of those factors are the quality of the RNA employed, the protocol of library preparation and the sequencing platform.

RNA-Seq sample size is generally and conveniently expressed in millions (M) of reads, and it is important because it limits the estimation of genes with low frequencies of expression. For example, if the sample size of a library is of 10 M reads, a gene with a relative frequency of expression of the inverse of that number, i.e., $1/(1 \times 10^7) = 1 \times 10^{-7}$ will have a probability of been detected with one or more reads of approximately of 0.63 However, under the same situation, a gene expressed at a frequency of only one in 100 millions, 1×10^{-8}, will have a probability of being detected with one or more reads of only ≈ 0.1, etc. Those calculations are assuming a binomial distribution for the number of reads, which is a reasonable model for this phenomenon.

A concern in this study was how much the variation in sample sizes among our RNA-Seq libraries could affect the estimation of diversity (ENL), specialization and locus specificity in our experiment. To address that concern we re-sampled our data assuming the binomial distribution, but using as sample size for each library the minimum number of reads obtained among the 179 libraries. All computations for this section are presented in `Box 1` in Appendix SI.5.1.

Date: February 11, 2024.

In the 179 RNA-Seq libraries analyzed here, sample size varied between a minimum of 10.33, and a maximum of 42.57, with an average of 16.83 millions of reads per library, thus there is a variation of more than 4 times more reads in the sample size of the largest with reference to the smallest library; $42.57/10.33 \approx 4.1$

To consider the worst case scenario, we obtained samples of each one of the 179 libraries, but with expected sample size equal to the one in the smallest library, i.e., we sampled only 10.33 M reads from each one of the original libraries. Those samples were taken by assuming the binomial distribution for each locus in each library, using as success probabilities the corresponding frequencies of each locus in each library. This procedure assume that the success probabilities are known, but introduces random variation by simulating the binomial realizations of each cell.

The expected sum of reads in the whole set of libraries in the sample is $179 \times 10.33 \approx 1,849.07$ M reads, and the realized value was 1,849.47 M reads, with a difference of only 28,278 reads between those sums. In the sample the number of reads per library varied between a minimum of 10.32, a maximum of 10.34 with a mean of 10.33 M reads per library, thus, the realized values were close to the expected number of reads per library, 10.33 M. The ratio of realized reduction in the whole sample sizes between the original data (3,012.85 M reads) and the one in the sample (1,849.5 M reads) was of $3012.85/1849.5 \approx 1.63$

We proceeded to compare the results of the parameters of interest from the analyses in the whole data set with those of the sample.

First, the differences in library diversity, measured as Effective Number of Loci (ENL) between the whole data and the sample were very small; the maximum difference was of 14 ENL, which is depreciable if we consider that the average ENL was 4,757 in the whole data set with an standard deviation of 1,245. In fact, the percent variations between the whole data and the sample varied between a minimum of -0.05% and a maximum of 0.31%, with an average of 0.12%. Furthermore, the Pearsons product-moment correlation between ENL in the whole data and the sample was practically equal to one ($\hat{r} = 0.9999975$). From this analysis we conclude that the differences in sample sizes between the libraries does not affect the estimation of transcriptome diversity.

Regarding the estimation of library specialization, the maximum difference between the whole data and the sample was -0.0009, which is very small if we consider that the average specialization in the whole data was 0.5057 with an standard deviation of 0.14; in fact, the percent variations between the whole data and the sample varied between a minimum of -0.70% and a maximum of -0.13%, with an average of -0.36%, and also the Pearsons product-moment correlation between the specialization values in the whole data and the samples was almost one ($\hat{r} = 0.9999964$). Thus, as in the case of transcriptome diversity, for library specialization we conclude that the differences in sample sizes between the libraries does not substantially alter the estimates of the parameter.

With reference to Locus Specificity (LS), this parameter is the one that could be more strongly affected by the sample sizes of the libraries, because at least for loci with small estimated frequencies of expression, the sample size of the libraries could produce an appreciable difference in estimates. The average LS in the whole data was 0.20129, with an standard deviation of 0.26, while the average LS in the sample was slightly larger, 0.20542 but with the same standard deviation of 0.26 In this case the Pearsons product-moment correlation between LS values in the whole data and the samples was large, but significantly smaller than one; $\hat{r} = 0.8790, \hat{r}^2 = 0.7727$. The 95% Confidence Interval (CI) for r was small, from 0.8767 to 0.8814, indicating that even with smaller sample sizes (as the ones employed in the samples), LS estimates are robust and biologically relevant.

To graphically appreciate the differences in diversity and specialization between the whole data and the sample, Figure 1 presents the plot of the corresponding points.

The circles plot in Figure 1 correspond to the values in the whole data frame, thus they are plot at exactly the same positions than the points presented in “**Figure 1**” in the main text of the paper, except that here we do not include the annotations per source and time of fruit development. The red points in

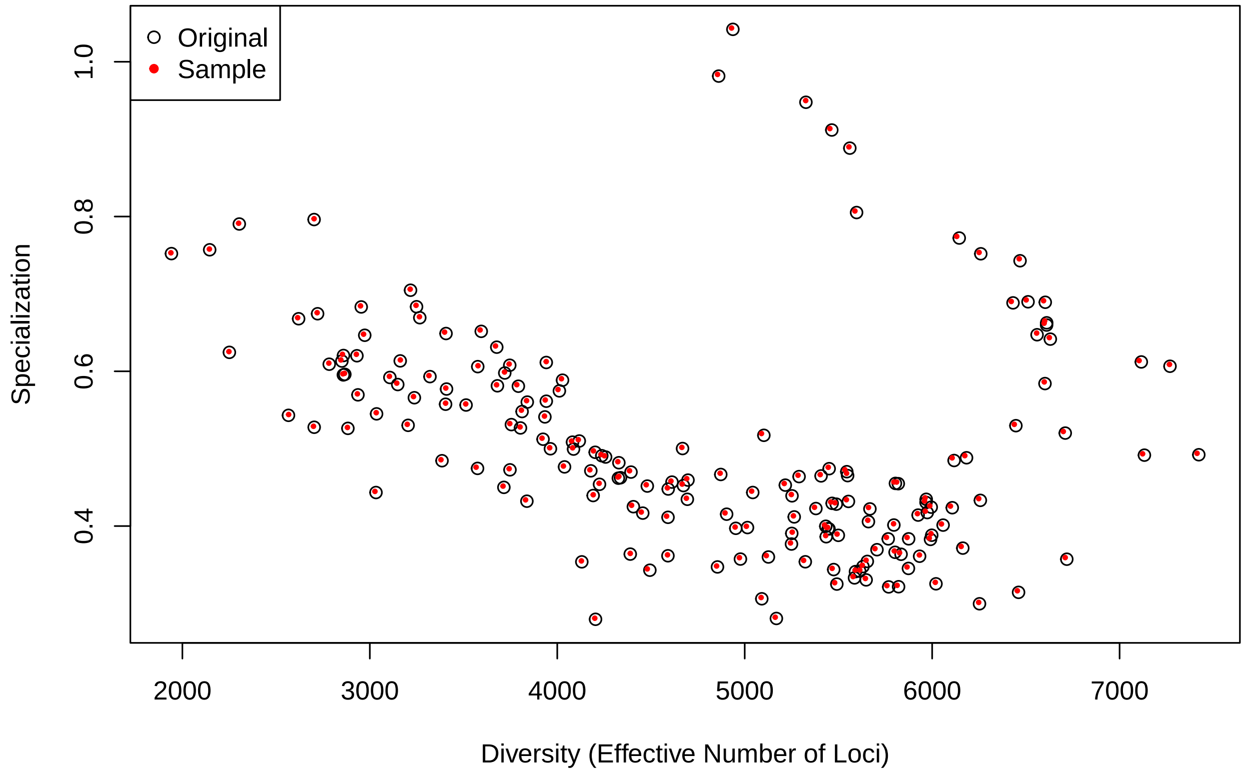

Figure 1. Results of estimates of diversity, as Effective Number of Loci (X-axis) $\times$ Library specialization (Y-axis) using the original data (circles) and the reduced sample (red points).

Figure 1 here were the ones estimated from the reduced sample, in which each one of the 179 libraries has only 10.33 million reads. As we can see in Figure 1 the points obtained from the whole data and the ones from the sample are almost in the same positions; the red points are always inside the circles, denoting a very small difference. Thus, the differences between the analysis in the whole data and the sample are not biologically relevant.

From analysis of sample size sensitivity presented here we conclude that the heterogeneity of sample sizes among libraries does not has strong effects over the estimates and thus all estimates obtained are robust and reliable.

SI.2. Global analysis per library

In the global analysis per library we study the results of diversity (ENL) and specialization using the 179 RNA-Seq libraries as experimental units. However, only fruit libraries are analyzed for those parameters to determine the effects of time and accession; the 4 libraries from siblings were excluded, given that the results are not comparable with the ones from fruits. Computations for this sections are presented in `Box 2` within Appendix SI.5.2.

The Analysis of Variance (ANOVA) of transcriptome diversity (ENL) as function of the factors accession (genotype) and time of fruit development –taken as factor in R, show that both sources of variation as well as its interaction are highly significant (p-value < 0.001). This means that the complexity of the transcriptomes, measured by ENL, is strongly affected by both, the genotype as well as the time of development and also that each accession presents particular values of ENL for each time of fruit development, given that the interaction term in the ANOVA was highly significant (p-value $= 0.000622$).

Residuals from the ANOVA show a normal distribution (p-value = 0.4227 in the Shapiro -Wilks test for normality), and the means of the model were kept for fitting linear models.

On the other hand, transcriptome specialization is also strongly affected by the genotype, the time of development and by the interaction of those two factors. The effects of those factors on specialization are stronger than for the case of ENL, which can be corroborated by the fact that the comparable values of the F statistic are larger than in the previous ANOVA (for ENL) and obviously the p-values are also much smaller for specialization than for ENL. In this case the test for normality of the residuals is rejected (p-value = 0.0003), due to a few outliers.

Taking together the ANOVAS for ENL and specialization, we can conclude that both, genotype and time of development, have a significant effect on those global transcriptome properties, a fact that suggest that the selection history of domesticated accessions from their wild ancestors, as well as the more recent artificial selection to obtain different domesticated genotypes has strongly modified gene expression.

To evaluate the effect of time as a continuous variable on ENL and specificity we fitted linear models to those variables, and the results are presented in in the main text in **Figure 2** for ENL and **Figure 3** for specificity, while R computations are in `Box 2` of Appendix SI.5.2.

For ENL the simple model "`enl = 6197 - 46 time`", presented in **Figure 2** of the main text, is highly significant (p-value $< 2.2 \times 10^{-16}$) and explains approximately 62% of the ENL variance as function of time ($R^2_{adj} = 0.6201$). This means that in average every day that pass after anthesis, the complexity of the transcriptome decreases in approximately 46 ENL for all genotypes.

In contrast with the diversity (ENL), transcriptome specialization does not follow a simple linear model, and to explain a substantial fraction of the variance of specialization as function of time it was needed to fit a polynomial model of degree 4, which is shown in **Figure 3** of the main text. That model explains approximately 47% of the variance of specialization as function of time ($R^2_{adj} = 0.4661$), and all its terms are highly significant.

SI.3. Analyses by genotype

R computations for this section are presented in boxes 3 to 7 in Appendix SI.5.3.

In the chili pepper experiment we have 179 libraries, 175 of them from fruits in development from 12 different sources (accessions or genotypes), at different time points of fruit's development. In principle, the experiment was designed as a complete factorial with two factors: Source:12 accessions of types Domesticated "D" (6), Wild "W" (4) and two Crosses "C" and 7 different time points of fruit development: 0, 10, 20, 30, 40, 50 and 60 Days After anthesis (DAA), in which each combination of the two factors was replicated twice.

However, one of the two replicates from the source "QC" at time 10 DAA failed, and tus such source includes only 13 libraries. On the other hand, fruits from three sources, "CW", "JE" and "AS" took more than 60 days to reach the fully mature status, and thus it was necessary to sample in times longer than 60 DAA; at 70 DAA for "CW", "JE" and "AS" and also at 80 DAA for "AS" (Table 1).

In this section we analyze only the genotype factor or "source", and with this aim all libraries with the same genotype are added by loci, giving a matrix of counts in which each column has counts from all the different times of fruit development and rows are loci. That matrix of counts represents gene expression for each one of the 12 genotypes during the whole fruit development process. Given that genotypes are classified by types: Domesticated "D", Wild "W" and Crosses "C", extra inferences can be obtained. R computations and results are presented in `Box 3` in the Appendix SI.5.3.

SI.3.1. **Diversity and Specialization.** Source diversity was estimated, as before, by the Effective Number of Loci, `enl`, while source specialization was estimated with `lib.spec`. **Figure 4** in the main text presents the scatterplot of diversity (`enl`) $\times$ specialization (`lib.spec`) in the 12 different sources, using

Table 1. Common name (**Name**), source key (**Source**), type (**Type**), total number of libraries (**Libraries**), total number of tags in millions; **Tags (M)**, structure of libraries within source (**Structure**) and maximum time sampled (**Max.**).

**Name**	**Source**	**Type**	**Libraries**	**Tags (M)**	**Structure**	**Max.**
F_1: QU × CM	QC	C	13	236	(2 rep. × 7 times)-1	60
Criollo de Morelos 334	CM	D	14	250	2 rep. × 7 times.	60
Piquín Coahuila	CO	W	14	232	2 rep. × 7 times.	60
F_1: CM x QU	CQ	C	14	279	2 rep. × 7 times.	60
Piquín Queretaro	QU	W	14	221	2 rep. × 7 times.	60
Piquín Sonora Red	SR	W	14	224	2 rep. × 7 times.	60
Serrano Tampiqueño 74	ST	D	14	205	2 rep. × 7 times.	60
Piquín Sonora Yellow	SY	W	14	221	2 rep. × 7 times.	60
Zunla-1	ZU	D	14	226	2 rep. × 7 times.	60
California Wonder	CW	D	16	294	2 rep. × 8 times.	70
Jalapeño Espinalteco	JE	D	16	274	2 rep. × 8 times.	70
Ancho San Luis	AS	D	18	296	2 rep. × 9 times.	80

different symbols and colors for each one of the three types. Here Table 2 presents the estimated values of the parameters and their means.

Table 2. Values of diversity, **enl**, specialization, **lib.spec**, as well as number of expressed loci, **Exp. Loci** by source, **Source** and type, **Type**.

**Source**	**Type**	**Tags (M)**	**Exp. Loci**	**enl**	**lib.spec**
CW	D	294	28,779	6,401	0.07103
AS	D	296	29,186	6,244	0.07160
JE	D	274	28,768	6,361	0.08278
ST	D	205	28,506	5,759	0.09170
ZU	D	226	28,857	5,478	0.09244
CM	D	250	28,778	5,975	0.09428
QC	C	236	29,246	6,160	0.09495
QU	W	221	29,027	5,915	0.10125
CO	W	232	28,787	5,776	0.10511
SY	W	221	29,596	6,344	0.11209
SR	W	224	29,702	6,296	0.11322
CQ	C	279	29,191	4,779	0.11417
Means	D	257	28,812	6,036	0.08397
	W	224	29,278	6,083	0.10792
	C	258	29,218	5,470	0.10456
	All	246	29,035	5,957	0.09538

Figure 2 presents a hierarchical tree (dendrogram) showing more clearly the differences in the space of diversity × specialization, already presented in **Figure 4** in the main text.

In Figure 2 we can see that the cross "CQ" is an outlier, being in an isolated position far away from the other sources. Also, the closest pair of sources are the wild accessions "SR" and "SY" –both collected from the same locality in the Mexican state of Sonora. This confirm that geographical closeness in wild accessions is reflected into global transcriptome likeness. The next closest entities in the dendrogram are the two domesticated genotypes "CW" and "AS", which share the phenotypic characteristic of having the largest fruits in all the collection of sources. Next in closeness are the pairs of wild ancestors "CO" and "QU", at the same height than the pair "CM" (D) and its direct descendent, the cross "QC". Later "JE" joins the pair "CW", "AS", forming a compact cluster of 3 domesticated sources. Then at height

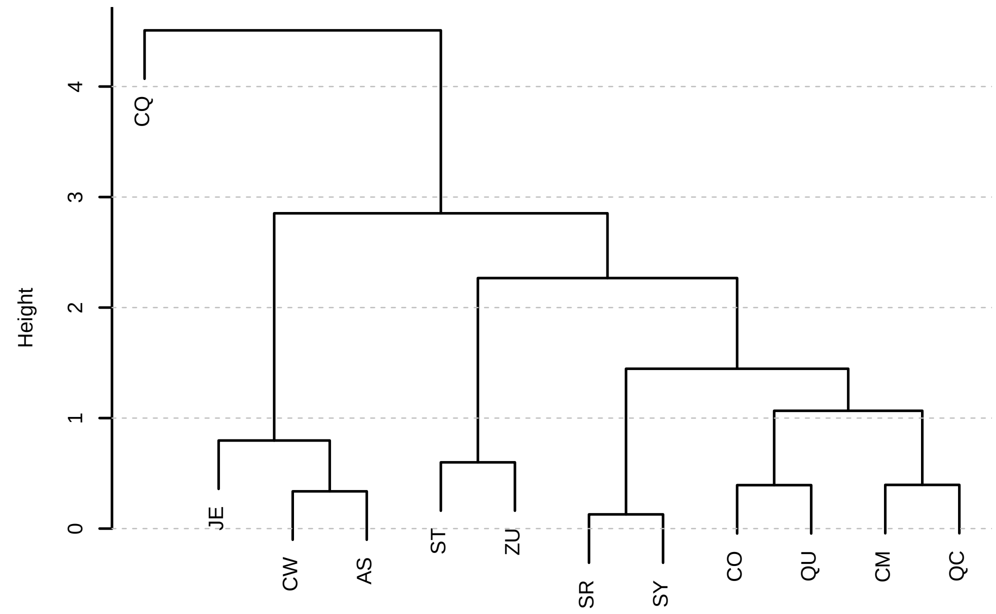

FIGURE 2. Dendrogram for genotypes obtained from the matrix of standardized distances in the space of Diversity (`s.enl`) × Specialization (`s.lib.spec`) by the complete method.

approximately 1.5 all four wild together with pair "CM", "QC" for a cluster, demonstrating that the domesticated "CM" is the closest to the ancestral *Capsicum.* Finally, at height slightly smaller than 3 all sources, except the outlier cross "CQ" are included into a single group.

In summary, the scatterplot in **Figure 4** on the main text and the dendrogram in Figure 2 delineate the panorama of diversity and specialization among the transcriptomes of different genotypes, allowing a better understanding of these informational properties in a representative sample of the fruits in the groups of domesticated, wild and crosses of this taxonomical group.

SI.3.2. **Study of locus specificity among sources.** Locus specificity, S_i, in the analysis performed by source, can vary between ≈ 0 –when the locus is "generalist" in the sense that it is expressed approximately at the same relative rate in all 12 sources, up to a maximum of $\log_2(12) = 3.584963$, when the locus is expressed in a single genotype. Defining relative S_i as $S_i/\log_2(12) = S_i/3.584963$ we obtain a measure of relative locus specificity that varies between 0 and 1 and which is easier to interpret. That quantity is denoted by "`rel.Si`" in the R code. R calculations and results for this section, which are focused on `rel.Si`, are presented in `Box 4` within Appendix SI.5.3.

Gene specificity, `rel.Si`, is trongly correlated with the maximum of gene expression per source, $\max_j(p_{ij})$. For example, in the data of this section that correlation was $\hat{r} = 0.9633$ Thus, it is reasonable to assign as source of gene specificity the accession in which the maximum is reached. For example, asume that for a given locus we have `rel.Si` = 1 –meaning that the locus was expressed in a single source. In that case the source of specificity is undoubtedly due to the accession where the maximum was obtained. By segregating gene specificities by source we are able to analyze separately `rel.Si` as function of the source.

Figure 3 presents the distribution of `rel.Si` in panel (A) and box plots with the distributions of the same parameter per source (accession) in panel (B). Panel (B) is also presented in the main text as **Figure 5**, but including extra annotations and ordering.

In panel (A) of Figure 3 we can appreciate that the distribution of `rel.Si` is highly biased toward small values, having a median of ≈ 0.04 and a mean of ≈ 0.16, with the highest frequency in the bar which includes values of `rel.Si` between 0 and 0.05 Interestingly, the last bar of this figure, which include

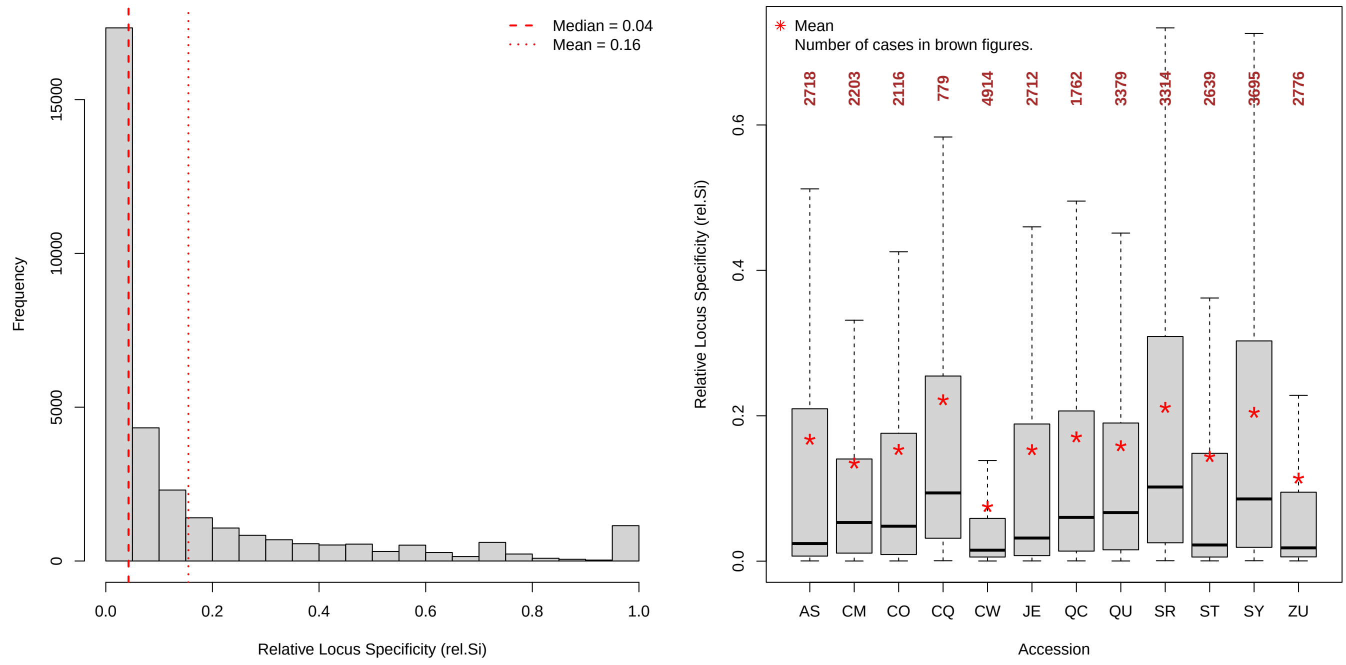

(A) General distribution (all sources).

(B) Segregated per source (accession).

FIGURE 3. Distribution of relative locus specificities (`rel.Si`).

the cases of `rel.Si` between 0.95 and 1 has a larger frequency than its immediate left hand side bars neighbors, which include values of `rel.Si` < 0.95

On the other hand, panel (B) of Figure 3 shows how the distributions of `rel.Si` classified by the accession at which the maximum expression is found are different in both, number of cases (brown vertical figures) and central tendency (medians as lines in the boxes, means as asterisks; see also **Figure 5** in the main text). In fact, the smallest mean of `rel.Si`, ≈ 0.08, is found in "CW", which at the same time has the largest number of `rel.Si`, 4,914 representing almost 15% of the total and, concurrently, "CW" is shown to be the source with less specialization and larger diversity in **Figure 4** in the main text (lower right hand size corner). In the same sense, the largest mean of `rel.Si`, ≈ 0.23, is found in the cross "CQ", which at the same time has the smallest number of `rel.Si`, 779, representing only 2% of the total, and also concurrently, "CQ" is the outlier in **Figure 4** in the main text, with the largest specialization and smaller diversity (upper left hand size corner in **Figure 4** in the main text); see other details in `Box 4`.

When classifying `rel.Si` by type ("D", "W" or "C"), the number of cases in "D" is of 17,962 loci (54%), with a mean `rel.Si` of ≈ 0.13, while "W" and "C", with 12,504 (38%) and 2,541 (8%) of the cases have both a mean `rel.Si` of ≈ 0.19, i.e., much less specific than the domesticated, "D", a fact that is also congruent with the results presented in **Figure 4** in the main text for library specialization.

The ANOVAS for mean `rel.Si` as function of accession and type are highly significant (p-value $< 1 \times 10^{-16}$), confirming that source strongly influence locus specificity (details in `Box 4`).

In panel (A) of Figure 3 we see that the relative specificity by source, `rel.Si`, includes the whole of the possible range, from `rel.Si = 0` up to `rel.Si = 1`. Genes with low relative specificity, say cases with `rel.Si` ≤ 0.05, are genes which are almost evenly expressed in all sources, and thus we label this group as "*generalist*" genes. The group of generalist genes has 17,333 elements, representing $\approx 53\%$ of the total of 33,007 loci studied ($17333/33007 \approx 0.5251$).

SI.3.2.1. *Gene Ontology (GO) enrichment analyses of generalist genes.* To obtain insights about the relevance of the set of generalist genes, we used the facilities for Gene Ontology enrichment analyses of our *Salsa* package. In such analyses Biological Process (BP), Molecular Functions (MF) and Cell Components (CC) are studied in 2×2 contingency tables comparing the frequency of occurrence of the genes in the target group (generalist genes) with the frequency of occurrence in the non-target group, and summarizing the results by the Odds Ratio (OR) of such tables. When OR is significantly larger than one it means that the target group in "enriched" in the studied category, while when the OR is significantly smaller than one the interpretation is that the target group is "depleted" of genes in the category. All analysis were performed controlling the False Discovery Rate, "FDR", at 1%, and we present tables for the relevant categories, including in each case the GO identifier of the category, which meaning can be clarified entering the GO identifier in the web site "GO search". In all tables with GO results we separately present sections of "enriched" and "depleted" cases separately. See computations in `Box 5` within Appendix SI.5.3.

Table 3 presents the results for GO Biological Process (BP) for generalist genes.

TABLE 3. GO Biological Processes significant (FDR 1%) in the set of "generalist" genes.

	Enriched (Odds Ratio > 4)	
**GO Identifier**	**Description**	**Odds Ratio**
GO:0040008	regulation of growth	Inf
GO:1901617	organic hydroxy compound biosynthetic process	16.13
GO:0019359	nicotinamide nucleotide biosynthetic process	13.44
GO:0072525	pyridine-containing compound biosynthetic process	8.07
GO:0016042	lipid catabolic process	6.28
GO:0071806	protein transmembrane transport	5.60
GO:0000105	histidine biosynthetic process	5.38
GO:0006333	chromatin assembly or disassembly	5.38
GO:0015684	ferrous iron transport	4.78
GO:0006417	regulation of translation	4.66
GO:0006826	iron ion transport	4.38
GO:0006888	ER to Golgi vesicle-mediated transport	4.04
	Depleted (Odds Ratio < 1/4)	
**GO Identifier**	**Description**	**Odds Ratio**
GO:0016998	cell wall macromolecule catabolic process	0.19 (5.26)
GO:0019684	photosynthesis, light reaction	0.17 (5.88)
GO:0046348	amino sugar catabolic process	0.11 (9.09)
GO:0006026	aminoglycan catabolic process	0.09 (11.11)

Table 3 show examples of the more relevan BP found. The first row shows that "*regulation of growth*" (GO:0040008) has the largest possible enrichment; the OR=Inf (infinite) means that all genes related with this process are present in the set of generalist genes. The 12 genes annotated into this process are mainly comprised by S-acyltransferases, and these proteins had been repeatedly related with fruit development and could be candidates for CRISPR/Cas-mediated genome editing into fruit species.

Other of the interesting categories in Table 3 is "*chromatin assembly or disassembly*" (GO:0006333), in which the 24 genes comprise histones, histone related proteins as well as 4 transcription factors annotated as telomere repeat-binding factors.

In general the 12 highly enriched (OR > 4) BP presented in Table 3 for the set of generalist genes make sense, because all those processes are relevant for fruit development and thus it is reasonable that the genes involved will have small specificity, been expressed without larger differences in all the 12 accessions during fruit development.

On the other hand, the 4 processes which present a significant reduction on genes (OR $< 1/4$; which inverse OR/4 is presented between parenthesis) are reasonable to be decreased in developing fruits. In particular, genes annotated in "*photosynthesis, light reaction*" (GO:0019684) are scarce in the generalist set of genes. Being the fruit a "sink" organ, photosynthetic activity is reduced trough its development, due to the conversion from chloroplast to chromoplasts which are the sites of carotenoid pigment synthesis and storage.

Table 4 presents the MF significant for the set of generalist genes.

TABLE 4. GO Molecular Functions significant (FDR 1%) in the set of "generalist" genes.

	Enriched (Odds Ratio > 5)	
**GO Identifier**	**Description**	**Odds Ratio**
GO:0016703	oxidoreductase activity, acting on single donors with incorporation of molecular oxygen, incorporation of one atom of oxygen (internal monooxygenases or internal mixed function oxidases)	17.95
GO:0045182	translation regulator activity	17.32
GO:0016423	tRNA (guanine) methyltransferase activity	8.51
GO:0009982	pseudouridine synthase activity	8.03
GO:0008176	tRNA (guanine-N7-)-methyltransferase activity	6.93
GO:0019200	carbohydrate kinase activity	5.67
GO:0015093	ferrous iron transmembrane transporter activity	5.03
	Depleted (Odds Ratio $< 1/5$)	
**GO Identifier**	**Description**	**Odds Ratio**
GO:0045156	electron transporter, transferring electrons within the cyclic electron transport pathway of photosynthesis activity	0.14 (7)
GO:0008061	chitin binding	0.14 (7)
GO:0016838	carbon-oxygen lyase activity, acting on phosphates	0.13 (8)
GO:0009916	alternative oxidase activity	0.12 (8)
GO:0008234	cysteine-type peptidase activity	0.11 (9)
GO:0010333	terpene synthase activity	0.10 (10)
GO:0016682	oxidoreductase activity, acting on diphenols and related substances as donors, oxygen as acceptor	0.10 (10)
GO:0004568	chitinase activity	0.06 (17)
GO:0016984	ribulose-bisphosphate carboxylase activity	0.06 (17)
GO:0080019	fatty-acyl-CoA reductase (alcohol-forming) activity	0.06 (17)
GO:0016168	chlorophyll binding	0.05 (20)
GO:0004097	catechol oxidase activity	0.00 (Inf)

In Table 4 we can see that there are 7 enriched MF with OR > 5 for generalist genes, i.e., cases where the number of generalist genes was at least 5 times greater in this target group of genes than the number expected at random. Within these MF the second more enriched, with OR $= 17.32$, has the identifier GO:0045182, corresponding to "*translation regulator activity*". This implies that genes related to the polypeptide synthesis at the ribosome are more than 17 times more abundant within the generalist genes than expected, underlying the importance of this process during fruit development.

On the other hand, the last row in Table 4, corresponding to the MF GO:0004097, "*catechol oxidase activity*", has a OR evaluated as 0 (inverse "Infinite"), meaning that none of the genes annotated in that MF are within the generalist set, verifying the absence of that functions in plants. Other 11 MF also show a depletion (OR $< 1/5$) for the set of generalist genes.

Table 5 shows the Cell Components (CC) enriched or depleted in the set of generalist genes.

In Table 5 the only enriched CC within generalist genes is GO:0005667, "*transcription factor complex*", with OR $= 3.34$ This means that the protein complex that associates with DNA by direct binding, or via

Table 5. GO Cell Components significant (FDR 1%) in the set of "generalist" genes.

	Enriched (Odds Ratio > 3)	
**GO Identifier**	**Description**	**Odds Ratio**
GO:0005667	transcription factor complex	3.34
	Depleted (Odds Ratio < 1/3)	
**GO Identifier**	**Description**	**Odds Ratio**
GO:0009579	thylakoid	0.28 (4)
GO:0009521	photosystem	0.25 (4)
GO:0009360	DNA polymerase III complex	0.22 (5)
GO:0009507	chloroplast	0.17 (6)
GO:0009536	plastid	0.17 (6)
GO:0005811	lipid particle	0.13 (8)
GO:0005853	eukaryotic translation elongation factor 1 complex	0.10 (10)

other DNA-binding proteins or complexes and regulating transcription is more than 3 times more frequent in generalist genes than the number expected. In total, there are 51 genes in this category ("*transcription factor complex*") within the set of generalist genes, and of these 51, there are 14 Transcription Factors (TF) which description is presented in Table 6.

Table 6. Description of the 14 Transcription Factors (from a total of 51 genes) which enriched for the Cell Component "GO:0005667" ("*transcription factor complex*"); see first row in Table 5.

**Gene id**	**Protein Description**
778	ABSCISIC ACID-INSENSITIVE 5-like protein 5
5507	transcription factor-like protein DPA isoform X1
6269	histone H2B.3-like
7856	transcription factor E2FB-like isoform X1
10145	auxin response factor 6-like
13320	E2F transcription factor-like E2FF isoform X1
15380	trihelix transcription factor GT-2-like
17143	transcription factor E2FC
18988	transcription factor MYB44-like
21966	LOB domain-containing protein 36-like
25387	transcription factor E2FA, partial
30253	transcription factor E2FB isoform X1
32973	E2F transcription factor-like E2FE
35136	transcription factor-like protein DPB isoform X1

Given that the 51 genes involved in the transcription factor complex play an important role in fruit development, we studied their normalized expression during this process in D and W accessions. That plot is presented in **Figure 6** of the main text, where its relevance is discussed.

SI.3.3. **Gene specificity by type.** In the previous section we studied transcriptome properties taking into account the 12 sources (accessions) without considering the time of fruit development. Here we carry on this process one step further by considering only two groups, the 6 domesticated (D) and the 4 wild (W) accessions. This implies that by grouping the fruit libraries into only two categories, D and W, we dismiss at this point the differences that exist both, among sources between type as well as between times of fruit development. R computations for this section are in `Box 7` in Appendix SI.5.3.

Table 7 presents the estimates of diversity and specialization of the analyses by type (D and W).

Table 7. Diversity and specialization results by type.

**Group** (`lib.name`)	**Million of tags** (`n.tags`)	**Exp. Loci** (`non.0`)	**E** (`enl`)	**Rel. E** (`rel.enl`)	δ (`lib.spec`)	**Rel.** δ (`rel.lib.spec`)
D	1,544	31,979	6,407	0.20037	0.02963	0.02963
W	898	31,729	6,253	0.19709	0.04137	0.04137

In Table 7 we can see that the number of expressed loci (**Exp. Loci**, `non.0` in `Box 5`) is larger in D, 31,979 than in W 31,729; i.e., 250 more genes detected as expressed in D compared with W. However, this difference could be due to the larger sample size of the D group which was obtained from a sample of 1,544, while for the W group the deep of sampling was of 898 (**Million tags** in Table 7), i.e., 1.72 times more reads in D compared with W.

On the other hand, the estimated number of effective loci, **E** in Table 7, as well as its relative measure, **Rel. E**, are not very different between the two groups. Nonetheless, that is not the case for the estimated group specialization, δ, which in R is denoted as "`lib.spec`". That parameter is 0.04137 in W, while it is almost 1.4 times smaller, $0.04137/0.02963 \approx 1.4$, in the D group. This indicates that when grouping the transcriptomes per type, the wild ancestors, W, have more specialized transcriptomes than the domesticated ones, D, a fact that was already observed in **Figure 4** in the main text when analyzing individual accessions.

SI.3.3.1. *Genes Exclusively Expressed (EE) in D or W accessions.* The analysis performed allows us to determine which genes were Exclusively Expressed (EE) in D or W accessions (see `Box 7`; objects related with EE genes include the letters "`FE`" in its name). EE genes are detected by having `rel.Si` $= 1$. A total of 2,012 genes were EE, representing $\approx 6\%$ of the total of genes expressed in fruit. Of these 2,012 genes, 1,131 ($\approx 56\%$) were expressed only in one or more of the D accessions, and 881 ($\approx 44\%$) were expressed only in one or more of the W accessions.

EE genes are interesting because the available evidence indicates that the domestication process modified its expression levels, fully suppressing its expression in one of the two groups (D or W), or alternatively, decreasing its expression below the detection level employed. However, this last alternative is unlikely, because the high statistical power of the experiments, which included hundreds of millions of reads from each type (see Table 7).

Figure 7 in the main text presents the plots of SEPs for the 191 ($191/1131 \approx 17\%$) EE in the D group and for the 157 ($157/881 \approx 18\%$) EE in the W group. The fact that only a small proportion of the EE genes have SEPs in each group is explained because, for many of those EE genes, expression was not detected in the whole set of D or W accessions and thus they are not included in the SEPs plot.

Also, **Table 1** in the main text presents the Transcription Factors (TF) found within the EE genes in the two groups.

SI.3.3.2. *GO analyses of the sets of Exclusively Expressed (EE).* Table 8 presents GO terms enriched (OR > 2, FDR 1%) in the sets of Exclusively Expressed (EE) genes by type, but in this case we segregate the GO categories in three subsets, the ones that appear in both, D and W types and the ones that appear only in one of the two types: D or W.

In Table 8, in the items enriched in both, D and W, we find in the first place *cysteine-type peptidase activity* (GO:0008234) that is enriched > 4.5 times in EE genes. The enrichment in this function implies that genes with a role in the catalysis of the hydrolysis of peptide bonds in a polypeptide chain are > 4.5 times more abundant in EE in both types, and coherently also "*proteolysis*", "*peptidase activity, acting on L-amino acid peptides*" and "*peptidase activity*", corresponding to GO:0006508, GO:0070011 and GO:0008233 are also enriched. The role of peptidase activity in fruit maturation in *Capsicum annuum*

Table 8. GO terms enriched (OR > 2, FDR 1%) in the sets of Exclusively Expressed (EE) genes.

			In both, D and W	
**GO**	**In**	**Aspect**	**Description**	**Odds ratio**
GO:0008234	both	MF	cysteine-type peptidase activity	D=4.78; W=4.56
GO:0006508	both	BP	proteolysis	D=2.67; W=3.19
GO:0070011	both	MF	peptidase activity, acting on L-amino acid peptides	D=2.77; W=2.63
GO:0008233	both	MF	peptidase activity	D=2.66; W=2.56
			Only in D	
**GO**	**In**	**Aspect**	**Description**	**Odds ratio**
GO:0031360	D	CC	intrinsic component of thylakoid membrane	D=24.64
GO:0009507	D	CC	chloroplast	D=8.59
GO:0009522	D	CC	photosystem I	D=6.66
GO:0044436	D	CC	thylakoid part	D=5.07
GO:0009521	D	CC	photosystem	D=4.73
GO:0003899	D	MF	DNA-directed RNA polymerase activity	D=2.70
GO:0098796	D	CC	membrane protein complex	D=2.60
GO:0034062	D	MF	RNA polymerase activity	D=2.49
GO:0032991	D	CC	macromolecular complex	D=2.13
GO:0043234	D	CC	protein complex	D=2.04
			Only in W	
**GO**	**In**	**Aspect**	**Description**	**Odds ratio**
GO:0019538	W	BP	protein metabolic process	W=2.03

has been previously reported, and our results here show that peptidase activity is highly relevant in both D and W accessions, and thus that such activity has not been strongly modified by domestication.

On the other hand, the CC and MF that are enriched only in D but not in W accessions ("Only in D" section in Table 8) involve mainly the thylakoid, chloroplast and photosystem. This enrichment in EE implies that the proteome adjustments that accompany chromoplast differentiation from chloroplasts during *Capsicum* fruit ripening, have been modified by domestication, given that these are enriched only in the D type of accessions.

SI.4. Analyses by time of development

In the previous sections we analyzed properties of the transcriptomes without taking into account the time of development of the fruit. Here we invert the point of view, we will study the properties of the transcriptomes for each time of fruit development, independently of the source (accession) from which the data arose. R computations for this section are in `Box 8` in the Appendix SI.5.4.

Table 9 presents transcriptome diversity and specialization by time of fruit development.

In Table 9 we can see that the sampling deepness of times from 0 to 60 DAA given in millions of tags (column "**Tags (M)**") was very high, having a median of more than 400 millions of reads per time. In contrast, times 70 and 80 DAA were estimated from much smaller libraries, having only 110 and 33 millions of tags, respectively. This is due to the fact that only 3 accessions has a time of fruit maturation larger than 60 DAA. However, those sample sizes are still large enough to estimate with precision transcriptome parameters.

Figure 8 in the main text presents the panorama of transcriptome diversity and specialization which data are shown in Table 9.

TABLE 9. Transcriptome diversity and specialization by time of fruit development.

Source	Tags (M)	Exp. Loci	enl	rel.enl	lib.spec	rel.lib.spec
T.0	406	30381	6804	0.22397	0.60653	0.19134
T.10	367	29834	6359	0.21314	0.31232	0.09852
T.20	405	30321	6230	0.20546	0.26310	0.08300
T.30	398	30263	5414	0.17889	0.29278	0.09236
T.40	437	29994	4316	0.14390	0.33736	0.10642
T.50	410	29594	4283	0.14473	0.35418	0.11173
T.60	392	29384	4168	0.14184	0.33893	0.10692
T.70	110	25716	3862	0.15017	0.28156	0.08882
T.80	33	22112	2775	0.12551	0.33822	0.10670

To clarify this panorama, Figure 4 presents the plot of Time (in DAA, X-axis) and diversity, "`enl`", in the Y-axis.

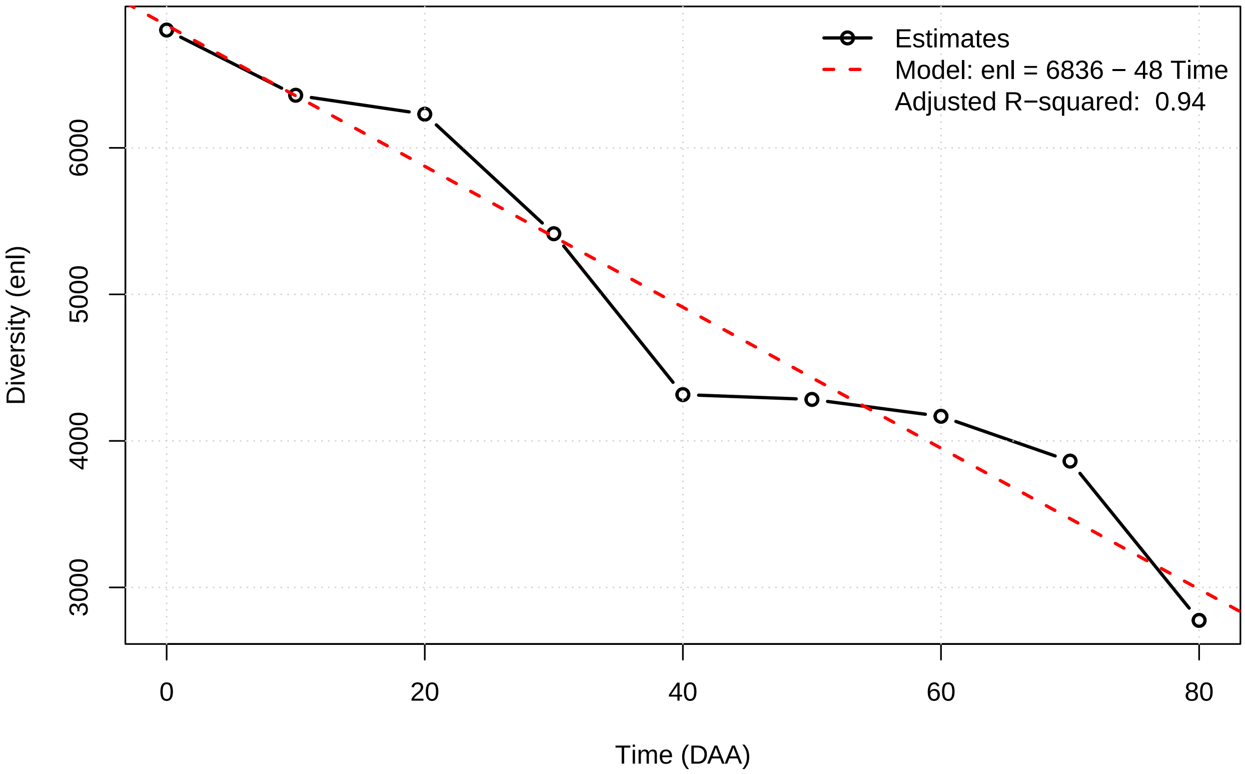

FIGURE 4. Plot of Time (in DAA, X-axis) and diversity, as Effective Number of Loci, `enl` (Y-axis) for all accessions.

In Figure 4 we can confirm what we previously observed in the general analysis of the data, i.e., that transcriptome diversity linearly decreases as function of time of fruit development, in this case without taking into account the accession of origin. The linear model, shown as a dashed red line in the graph, explains 94% of the variation of diversity as function of time, and implies that in average each day of fruit development decreases in approximately 48 Effective Number of Loci (`enl`) the diversity of the fruit transcriptome. Interestingly, this fact is also valid for the largest times of development, 70 and 80 DAA, which were explored only in the accessions with an extended time of fruit development, CW, AS and JE, of which CW and AS have the largest fruit sizes in the collection.

Figure 5 shows the plot of Time (in DAA, X-axis) and specialization, as relative specialization, `rel.lib.spec` (Y-axis) independently of the accession of origin.

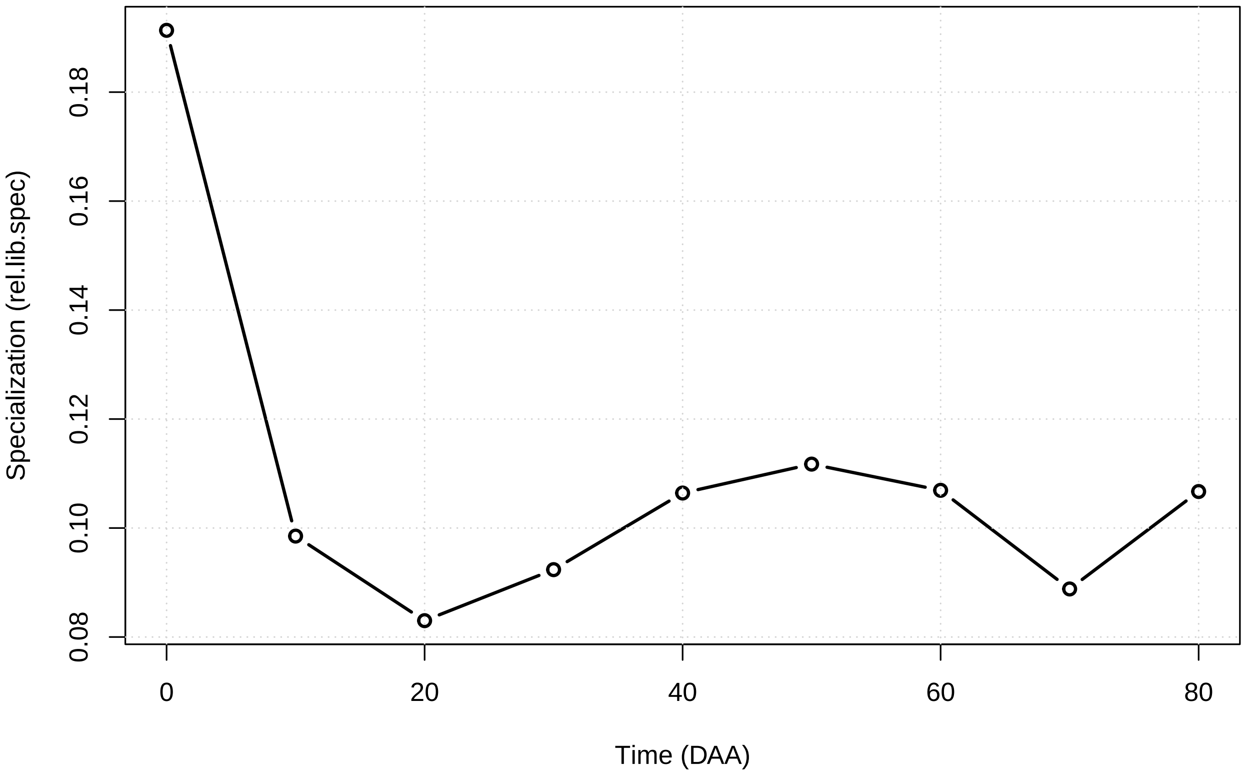

FIGURE 5. Plot of Time (in DAA, X-axis) and specialization, as relative specialization, `rel.lib.spec` (Y-axis) for all accessions.

In Figure 5 we can see how the behavior of the specialization, `rel.lib.spec`, as function of time of fruit development does not presents a linear relationship, as was the case for diversity. Specialization is very high at the stage of mature flower, 0 DAA, and then it decreases to reach its global minimum at 20 DAA, then it steadily increases up to 50 DAA, where it has a local maximum to further decrease until 70 DAA, and finally has an increase from 70 to 80 DAA. Because only one accession, the bell type AS, was sampled at 80 DAA, it is possible that the suden increase of specialization observed from 70 to 80 DAA will be due to genes expressed mainly in that large fruit accession of long fruit development cycle.

SI.4.1. **Highly specific genes per time of fruit development.** Figure 6 presents the distributions, as box plots, of locus specificity for each one of the 9 times of fruit development studied. Figure 6 is also presented as **Figure 9** in the main text.

In Figure 6 we can see that at 0 DAA the mean specificity of the loci, plot as a red asterisk, is much higher than in all the other times of fruit development. This makes sense, because in fact all times larger than 0 DAA are for fruits, while at 0 DAA we have a different organ, and it is logical that a large group of genes will be expressed preferentially (specifically) in the flower. A total of 986 genes show an specificity ≥ 0.9 at 0 DAA, while the numbers of highly specific genes are much smaller in the other stages of development. This is also influenced by the fact that the largest percentage of gene specificities, 22.6, occurs also at this stage.

On the other hand, the stages with less number and means of specific genes occur at 70 and 80 DAA (last two box plots in Figure 6), an interesting fact if we take into account that those two times were sampled only in three of the 12 accessions.

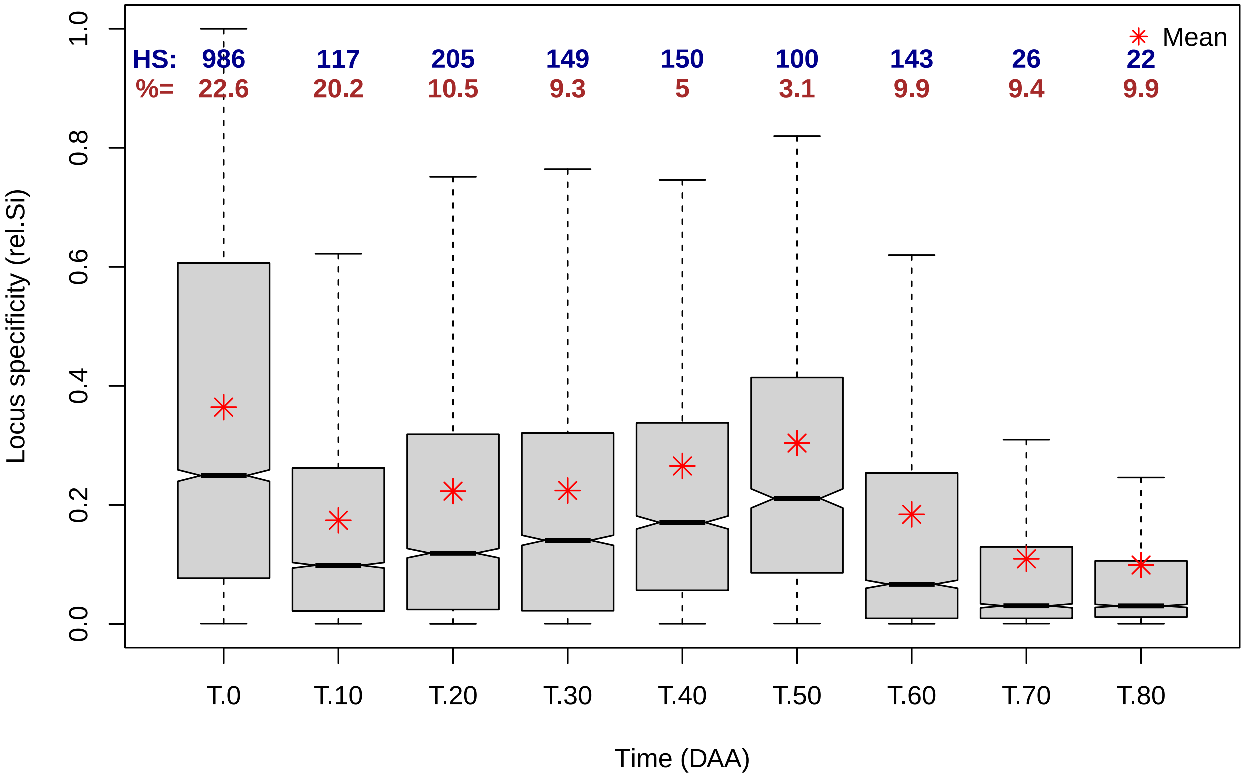

FIGURE 6. Distributions of `rel.Si` per time of development. Blue numbers (**HS:**) give the number of loci with `rel.Si` > 0.9, i.e., highly specific loci in each time. Brown numbers (%=) give the percentage of the total number cases per time of development (% of the total of 33,007 loci).

SI.4.1.1. *GO enrichment in highly specific genes per fruit development stages.* To have enough statistic power to perform fruitful GO enrichment analyses we must have enough genes in the target groups. With this aim we divided fruit development in three stages, first, the mature flower at 0 DAA, which has 986 highly specific, `rel.Si` > 0.9, second the growing stages, from 10 up to 40 DAA, which have $117 + 205 + 149 + 150 = 621$ genes with that characteristic and, finally, genes with high specificity in the maturity stage, the $100 + 143 + 26 + 22 = 291$ that have `rel.Si` > 0.9 in times 50 to 60 DAA (see Figure 6).

With those genes as target groups, we performed the GO enrichment analyses which are summarized in tables 10, 11 and 12 for the highly specific genes in the flower, growing and maturity stages, respectively.

The collection of MF, BP and CC terms enriched for genes with high specificity at 0 DAA in Table 10 show, has expected, terms that are more characteristic of the mature flower than from later stages in fruit development. In particular, the ones in the rows with Odds Ratios > 6, corresponding in decreasing OR order to "*solute:cation antiporter activity*" GO:0015298, "*pectinesterase activity*" GO:0030599, "*cell wall modification*" GO:0042545, "*antiporter activity*" GO:0015297, "*response to auxin*" GO:0009733 and "*polygalacturonase activity*" GO:0004650, are more characteristic of the flower than of posterior fruit stages of development.

In Table 11, for the enriched terms in genes highly specific during fruit growing stages, we see BP and CC terms mainly linked with photosynthesis and MF related to peptidase activity.

Table 12 presents results of GO enrichment analysis of genes highly specific to maturity (Times 50 DAA or more).

Table 10. GO enrichment analysis of highly specific genes in flower (Time 0 DAA; `Box 8`).

GO	Aspect	Description	Odds ratio
GO:0015298	MF	solute:cation antiporter activity	8.53
GO:0030599	MF	pectinesterase activity	7.64
GO:0042545	BP	cell wall modification	7.31
GO:0015297	MF	antiporter activity	7.10
GO:0009733	BP	response to auxin	6.85
GO:0004650	MF	polygalacturonase activity	6.67
GO:0009725	BP	response to hormone	5.51
GO:0071555	BP	cell wall organization	5.10
GO:0015291	MF	secondary active transmembrane transporter activity	4.35
GO:0005618	CC	cell wall	4.28
GO:0071554	BP	cell wall organization or biogenesis	3.92
GO:0052689	MF	carboxylic ester hydrolase activity	3.55
GO:0030312	CC	external encapsulating structure	3.40
GO:0004857	MF	enzyme inhibitor activity	3.19
GO:0030234	MF	enzyme regulator activity	2.98
GO:0008289	MF	lipid binding	2.60
GO:0098772	MF	molecular function regulator	2.47
GO:0008234	MF	cysteine-type peptidase activity	2.25
GO:0004553	MF	hydrolase activity, hydrolyzing O-glycosyl compounds	2.17
GO:0016798	MF	hydrolase activity, acting on glycosyl bonds	2.13

Table 11. GO enrichment analysis of highly specific genes in growing (Times 10 to 40 DAA; `Box 8`).

GO	Aspect	Description	Odds ratio
GO:0019684	BP	photosynthesis, light reaction	5.87
GO:0008234	MF	cysteine-type peptidase activity	4.85
GO:0009579	CC	thylakoid	4.56
GO:0034357	CC	photosynthetic membrane	4.49
GO:0009521	CC	photosystem	4.42
GO:0015979	BP	photosynthesis	3.87
GO:0006508	BP	proteolysis	3.14
GO:0070011	MF	peptidase activity, acting on L-amino acid peptides	3.10
GO:0098796	CC	membrane protein complex	2.35

In Table 12 we can appreciate how the enriched terms are related mainly with processes important in the late stage of fruit maturation.

It is interesting to note that the only MF common to Tables 10, 11 and 12 corresponds to GO:0008234 ("*cysteine-type peptidase activity*"). Table 13 presents the protein identifiers and description of 6 of the genes with clear annotation in this category.

Some of the genes presented in Table 13, and annotated as playing an important role in "*cysteine-type peptidase activity*" could also be involved in salt and osmotic induced leaf senescence in *Capsicum*.

It is important to remark that, even when a set of genes were found as enriched in the three stages of fruit development, as the ones in Table 13 for the MF "*cysteine-type peptidase activity*", one needs to examine the expression of those genes at different times of development to reach biological conclusions. As an example of this, Figure 7 shows the Standardized Expression Profiles (SEPs) for the 6 genes in Table 13.

Table 12. GO enrichment analysis of genes highly specific to maturity (Times 50 DAA or more; `Box 8`).

GO	Aspect	Description	Odds ratio
GO:0009916	MF	alternative oxidase activity	30.40
GO:0016682	MF	oxidoreductase activity, acting on diphenols and related substances as donors, oxygen as acceptor	17.91
GO:0009522	CC	photosystem I	12.70
GO:0009579	CC	thylakoid	7.61
GO:0044436	CC	thylakoid part	7.61
GO:0009521	CC	photosystem	7.47
GO:0034357	CC	photosynthetic membrane	7.02
GO:0008234	MF	cysteine-type peptidase activity	6.80
GO:0098796	CC	membrane protein complex	5.11
GO:0032991	CC	macromolecular complex	3.87
GO:0043234	CC	protein complex	3.76
GO:0070011	MF	peptidase activity, acting on L-amino acid peptides	3.40
GO:0006508	BP	proteolysis	3.30
GO:0008233	MF	peptidase activity	3.27

Table 13. Well identified genes within the ones enriched for GO:0008234 ("*cysteine-type peptidase activity*") in Tables 10, 11 and 12.

id	Protein Id	Description
491	XP_016548765.1	fasciclin-like arabinogalactan protein 12
5144	XP_016570537.1	senescence-specific cysteine protease SAG39-like
19052	XP_016559739.1	senescence-specific cysteine protease SAG39-like
8705	XP_016579481.1	ervatamin-B
20327	XP_016552838.1	putative clathrin assembly protein At1g03050
32945	XP_016568442.1	kinesin-like protein NACK1 isoform X1

Figure 7 shows how the 6 genes identified with the MF "*cysteine-type peptidase activity*" have an expression pattern with a global and clear maximum at the mature flower (0 DAA). The 95% CI for the mean expression at each time of development (vertical lines) are overlap for the three types of accessions, indicating that there is not strong differences of expression in this group of genes by type of accession.

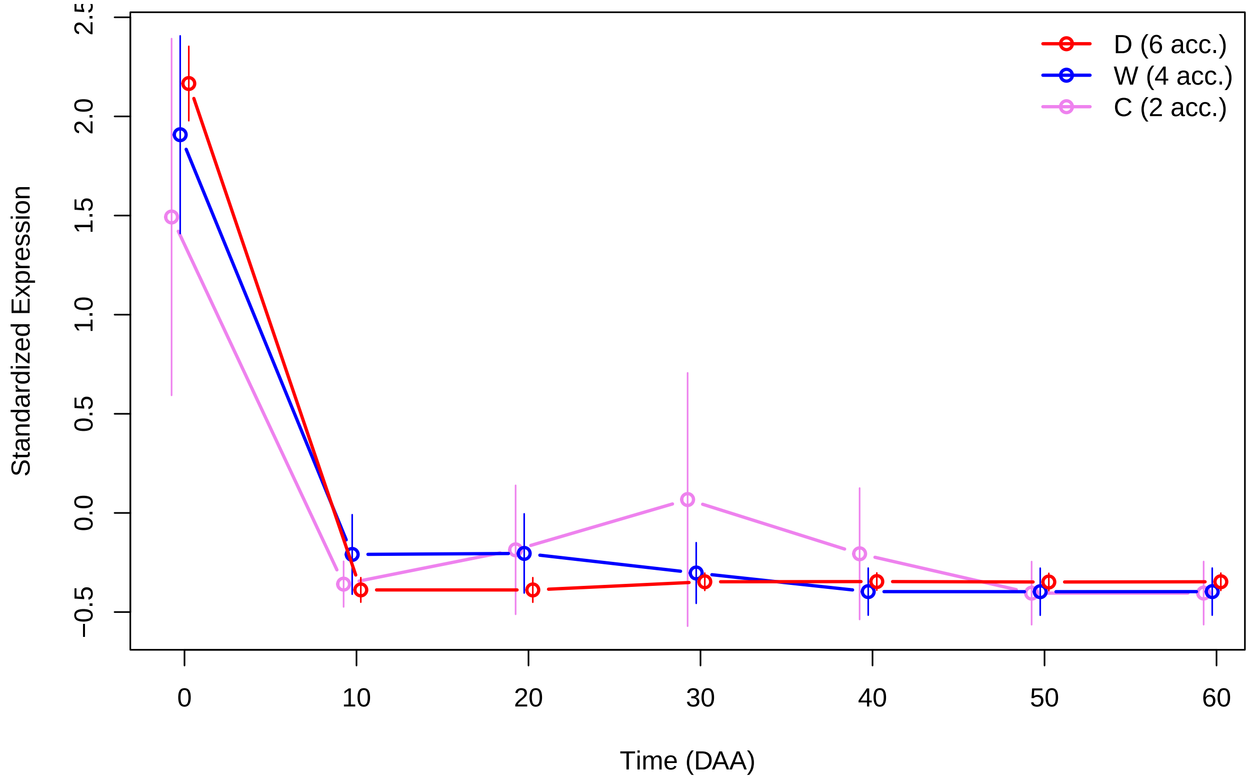

FIGURE 7. SEPs plot for the 6 genes in Table 13.

SI.5. Appendix: Boxes with R code

To replicate computations presented here you need to instal the R packages "*infoRNAseq*" and "*Salsa*" (both accessed on 5 February 2024). Consecutive text boxes present the computations for each sections and are order-depending, i.e., they must be performed in the same order than presented here. All objects produced as results of the R computations are available upon request in the binary R object "`ObjectsForSupplementary.RData`".

SI.5.1. **Computations for "Sample size sensitivity analysis".**

```
------------------------------------------------------------------------------------------
# Box 1. Sensitivity analysis.
# Load libraries
> library(infoRNAseq)
> library(Salsa)

# Perform general analysis to detect sample size per library
> all.ca.c <- est.all(ca.c)
> names(all.ca.c) # A list with all results and components:
[1] "diversity" "spec"      "locus"     "freq"
> names(all.ca.c$diversity) # Names of components of that data.frame
[1] "lib.name"   "n.tags"     "lib.length" "non.0"      "H"          "rel.H"
[7] "enl"        "rel.enl"
> nrow(all.ca.c$spec) # Total number of libraries in the experiment
[1] 179
> summary(all.ca.c$spec$n.tags) # Statistics for sample sizes in the 179 libraries
    Min.  1st Qu.   Median     Mean  3rd Qu.     Max.
10332232 14980394 16442302 16831551 18213232 42565757
> sd(all.ca.c$spec$n.tags) # Standard deviation
[1] 3315949
> summary(all.ca.c$spec$n.tags/1e6) # Sample sizes in the 179 libraries in millions
   Min. 1st Qu.  Median    Mean 3rd Qu.    Max.
  10.33   14.98   16.44   16.83   18.21   42.57

# We are going to create a SAMPLE of the original data "ca.c", assuming for each
# cell in the sample a binomial distribution with the frequencies estimated in
# the original data, but with size of 10,332,232 reads (the minimum library size)

# First data with 0 reads for all loci in all libraries:
> sam.ca.c <- data.frame(matrix(0, nrow=nrow(ca.c), ncol=ncol(ca.c),
  dimnames=list(attributes(ca.c)$row.names, names(ca.c))))

# And perform the sampling in two loops
set.seed(1959+2026) # To make reproducible the process.
n.min <- 10332232
temp.rows <- 35883
for(j in 1:179){
for(i in 1:temp.rows){
if(all.ca.c$freq[i,j]>0){
sam.ca.c[i,j] <- rbinom(n=1, size=n.min, prob=all.ca.c$freq[i,j])
}
}
```

```
}

> sum(sam.ca.c) # Must be "close" to 179*10332232 and smaller than sum(ca.c)
[1] 1849497806
> 179*10332232
[1] 1849469528
> 1849497806-1849469528 # Difference observed with expected
[1] 28278
> sum(ca.c)/sum(sam.ca.c) # How many times smaller the sample compared with original
[1] 1.629009

> summary(apply(sam.ca.c, 2, sum)) # Variability in sample sizes
    Min.  1st Qu.   Median     Mean  3rd Qu.     Max.
10322522 10330538 10332336 10332390 10334438 10342543

> summary(apply(sam.ca.c, 2, sum)/1e6) # Variability in sample sizes (millions)
   Min. 1st Qu.  Median    Mean 3rd Qu.    Max.
  10.32   10.33   10.33   10.33   10.33   10.34

# Now let's obtain the results in the sample:
> sam.all.ca.c <- est.all(sam.ca.c) # Obtain the results in the sample.

> summary(all.ca.c$diversity$enl) # Summary of diversity (ENL) in original
   Min. 1st Qu.  Median    Mean 3rd Qu.    Max.
   1942    3774    4904    4757    5736    7423
> summary(sam.all.ca.c$diversity$enl) # Summary of diversity (ENL) in sample
   Min. 1st Qu.  Median    Mean 3rd Qu.    Max.
   1939    3765    4895    4752    5725    7413
# Let's make a summary of the differences in ENL (original-sample)
> summary(all.ca.c$diversity$enl - sam.all.ca.c$diversity$enl)
   Min. 1st Qu.  Median    Mean 3rd Qu.    Max.
 -2.532   3.542   5.542   5.689   7.992  13.795
# And a summary of the percentages (original-sample)/original:
> summary(100*(all.ca.c$diversity$enl - sam.all.ca.c$diversity$enl)/all.ca.c$diversity$enl)
    Min.  1st Qu.   Median     Mean  3rd Qu.     Max.
-0.04822  0.07767  0.12530  0.11876  0.15417  0.30695
# Also a measure of the linear correlation between ENL in original and sample
> cor.test(all.ca.c$diversity$enl, sam.all.ca.c$diversity$enl)

Pearson's product-moment correlation

data:  all.ca.c$diversity$enl and sam.all.ca.c$diversity$enl
t = 5921.4, df = 177, p-value < 2.2e-16
alternative hypothesis: true correlation is not equal to 0
95 percent confidence interval:
 0.9999966 0.9999981
sample estimates:
      cor
0.9999975

# Let's now investigate what happened with the estimates of specialization.
> summary(all.ca.c$spec$lib.spec) # In the original data
```

```
   Min. 1st Qu.  Median    Mean 3rd Qu.    Max.
 0.2795  0.4013  0.4726  0.5057  0.5926  1.0419
> summary(sam.all.ca.c$spec$lib.spec) # In the reduced sample
   Min. 1st Qu.  Median    Mean 3rd Qu.    Max.
 0.2809  0.4028  0.4741  0.5074  0.5940  1.0436
> summary(all.ca.c$spec$lib.spec - sam.all.ca.c$spec$lib.spec) # original - sample
      Min.    1st Qu.     Median       Mean    3rd Qu.       Max.
-0.0034099 -0.0018480 -0.0016801 -0.0016778 -0.0013860 -0.0009112
# And a summary of the percentages (original-sample)/original:
> summary(100*(all.ca.c$spec$lib.spec - sam.all.ca.c$spec$lib.spec)/all.ca.c$spec$lib.spec)
   Min. 1st Qu.  Median    Mean 3rd Qu.    Max.
-0.7035 -0.4321 -0.3441 -0.3561 -0.2584 -0.1260
# Also a measure of the linear correlation in the original and sample
> cor.test(all.ca.c$spec$lib.spec, sam.all.ca.c$spec$lib.spec)

Pearson's product-moment correlation

data:  all.ca.c$spec$lib.spec and sam.all.ca.c$spec$lib.spec
t = 4953.9, df = 177, p-value < 2.2e-16
alternative hypothesis: true correlation is not equal to 0
95 percent confidence interval:
 0.9999952 0.9999973
sample estimates:
      cor
0.9999964

# Now examine differences in relative locus specificities
> summary(all.ca.c$locus$rel.Si)
   Min. 1st Qu.  Median    Mean 3rd Qu.    Max.
0.00000 0.01487 0.08695 0.20129 0.30050 1.00000
> summary(sam.all.ca.c$locus$rel.Si)
   Min. 1st Qu.  Median    Mean 3rd Qu.    Max.
0.00000 0.01406 0.08362 0.20542 0.30947 1.00000
> summary(all.ca.c$locus$rel.Si - sam.all.ca.c$locus$rel.Si) # Differences
      Min.    1st Qu.     Median       Mean    3rd Qu.       Max.
-0.4483384 -0.0159797 -0.0009894 -0.0041314 -0.0000639  1.0000000
> cor.test(all.ca.c$locus$rel.Si, sam.all.ca.c$locus$rel.Si)

Pearson's product-moment correlation

data:  all.ca.c$locus$rel.Si and sam.all.ca.c$locus$rel.Si
t = 349.24, df = 35881, p-value < 2.2e-16
alternative hypothesis: true correlation is not equal to 0
95 percent confidence interval:
 0.8766553 0.8813596
sample estimates:
      cor
0.8790289

# Finally, to visually corroborate that the differences are very small and of no
# biological consequences, let's make a plot of all libraries with the original and
# sample values:
```

```
# Plot values (presented as Figure 1 in this document):
plot(all.ca.c$diversity$enl, all.ca.c$spec$lib.spec,
xlab="Diversity (Effective Number of Loci)",
ylab="Specialization")
# Plot the values in the sample as small red points.
points(sam.all.ca.c$diversity$enl, sam.all.ca.c$spec$lib.spec, pch=20, cex=0.5, col="red")
legend("topleft", legend=c("Original", "Sample"), pch=c(1, 20), col=c("black", "red"))
-----------------------------------------------------------------------------------------
```

SI.5.2. **Computations for "Global analysis per library".**

```
-----------------------------------------------------------------------------------------
# Box 2. Global analysis per library
# Note: Previously in Box 1 we created ca.fru.all (general results with all libraries).
# Here we perform the general analyses of diversity and specialization.

# Isolate results to be analyzed (do not include siblings, only fruits)
> ca.fru.all.enlXspec <- data.frame(ca.fru.all$diversity[,c(1,7)],
   lib.spec=ca.fru.all$spec$lib.spec)
> ca.fru.all.enlXspec <- cbind(ca.fru.all.enlXspec, ca.fru.def[,2:5])
> ca.fru.all.enlXspec$time.f <- as.factor(ca.fru.all.enlXspec$time)

# ANOVA for library diversity (enl)
> aov.enl.ca.fru.all.enlXspec <- aov(enl ~ acc*time.f, data=ca.fru.all.enlXspec)
> summary(aov.enl.ca.fru.all.enlXspec)
            Df    Sum Sq  Mean Sq F value   Pr(>F)
acc         11  11949640  1086331   3.670 0.000267 ***
time.f       8 190826963 23853370  80.578  < 2e-16 ***
acc:time.f  68  42043708   618290   2.089 0.000622 ***
Residuals   87  25754436   296028
> shapiro.test(aov.enl.ca.fru.all.enlXspec$residuals)
Shapiro-Wilk normality test
data:  aov.enl.ca.fru.all.enlXspec$residuals
W = 0.9918, p-value = 0.4227
# Model tables (to be used later in model)
> mt.aov.enl.ca.fru.all.enlXspec <- model.tables(aov.enl.ca.fru.all.enlXspec, type="means")

# Obtain the ANOVA for library specificity:
> aov.spec.ca.fru.all.enlXspec <- aov(lib.spec ~ acc*time.f, data=ca.fru.all.enlXspec)
> summary(aov.spec.ca.fru.all.enlXspec)
            Df Sum Sq Mean Sq F value   Pr(>F)
acc         11 0.6440 0.05855  19.465  < 2e-16 ***
time.f       8 1.7478 0.21848  72.638  < 2e-16 ***
acc:time.f  68 0.8159 0.01200   3.989 1.17e-09 ***
Residuals   87 0.2617 0.00301
> shapiro.test(aov.spec.ca.fru.all.enlXspec$residuals)
Shapiro-Wilk normality test
data:  aov.spec.ca.fru.all.enlXspec$residuals
W = 0.96659, p-value = 0.0003299
# Residuals are not normal, however will use this model
# however, that is due to a few outliers; see
```

```
> plot(aov.spec.ca.fru.all.enlXspec)
# Model tables to be used later in plot:
> mt.aov.spec.ca.fru.all.enlXspec <- model.tables(aov.spec.ca.fru.all.enlXspec, type="means")

# Operations to obtain models.
# For enl:
> all.ca.c.f.div <- all.ca.c$diversity
> all.ca.c.f.div$time <- as.integer(substring(all.ca.c.f.div$lib.name, 3, 4))
Warning message:
NAs introduced by coercion
> nrow(all.ca.c.f.div[!is.na(all.ca.c.f.div$time),])
[1] 175
> all.ca.c.f.div <- all.ca.c.f.div[!is.na(all.ca.c.f.div$time),]
> all.ca.c.f.div$acc <- substring(all.ca.c.f.div$lib.name, 1, 2)
> all.ca.c.f.div$type <- ""
for(i in 1:175){
all.ca.c.f.div$type[i] <- acc$acc.type[all.ca.c.f.div$acc[i]==acc$acc.key]
}
> summary(lm(enl ~ time, data=all.ca.c.f.div))
Coefficients:
            Estimate Std. Error t value Pr(>|t|)
(Intercept) 6197.033    104.415   59.35   <2e-16 ***
time         -45.689      2.706  -16.88   <2e-16 ***
Residual standard error: 768.6 on 173 degrees of freedom
Multiple R-squared:  0.6223,Adjusted R-squared:  0.6201
F-statistic:   285 on 1 and 173 DF,  p-value: < 2.2e-16

> lm.enlXtime.all.ca <- lm(enl ~ time, data=all.ca.c.f.div)
> pr.lm.enlXtime.all.ca <- as.data.frame(predict.lm(lm.enlXtime.all.ca,
   newdata=data.frame(time=c(0:80)), interval="prediction", level=0.95))

# pch and col for plot:
all.ca.c.f.div$pch <- NA
all.ca.c.f.div$pch[all.ca.c.f.div$type=="D"] <- 16 # circle
all.ca.c.f.div$pch[all.ca.c.f.div$type=="W"] <- 15 # square
all.ca.c.f.div$pch[all.ca.c.f.div$type=="C"] <- 18 # diamond
all.ca.c.f.div$col <- ""
all.ca.c.f.div$col[all.ca.c.f.div$type=="D"] <- "red"
all.ca.c.f.div$col[all.ca.c.f.div$type=="W"] <- "blue"
all.ca.c.f.div$col[all.ca.c.f.div$type=="C"] <- "purple"

# Plot (presented as "Figure 2" in main text:
plot(all.ca.c.f.div$time, all.ca.c.f.div$enl, pch=all.ca.c.f.div$pch, col=all.ca.c.f.div$col,
xlab="Time (DAA)", ylab="Diversity (Effective Number of Loci)")
points(seq(0, 80, by=10), tapply(all.ca.c.f.div$enl, all.ca.c.f.div$time, mean), pch=10,
cex=1.5)
abline(lm.enlXtime.all.ca, lwd=2.5)
points(c(0:80), pr.lm.enlXtime.all.ca$lwr, type="l", lty=2, col="grey", lwd=2)
points(c(0:80), pr.lm.enlXtime.all.ca$upr, type="l", lty=2, col="grey", lwd=2)
#, bty="n"
legend("topright", legend=c("Domesticated", "Wild", "Cross", "Mean per time"),
pch=c(16, 15, 18, 10), col=c("red", "blue", "purple", "black"))
```

```
legend("bottomleft", bty="n", legend=c("Model: ENL = 6,197 - 46 Time",
"(R^2 = 0.62)", "95% CI for predictions."), lty=c(1, 1, 2), lwd=c(2.5, 0, 2),
col=c("black", "white", "grey"))

# Modeling specificity (final model lm4.all.ca.c.spec)
> all.ca.c.spec <- all.ca.c$spec[,c(1,5:6)]
> all.ca.c.spec$time <- as.integer(substring(all.ca.c.spec$lib.name, 3,4))
Warning message:
NAs introduced by coercion
# Takes out not valid times:
> all.ca.c.spec <- all.ca.c.spec[!is.na(all.ca.c.spec$time),]
> all.ca.c.spec$acc <- substring(all.ca.c.spec$lib.name, 1, 2)
> nrow(all.ca.c.spec)
[1] 175
# Obtain type:
> all.ca.c.spec$type <- ""
for(i in 1:175){
all.ca.c.spec$type[i] <- acc$acc.type[acc$acc.key==all.ca.c.spec$acc[i]]
}
c("Domesticated", "Wild", "Cross", "Mean per time"), pch=c(16, 15, 18, 10),
 col=c("red", "blue", "purple", "black"))
> all.ca.c.spec$pch <- NA
> all.ca.c.spec$pch[all.ca.c.spec$type=="D"] <- 16
> all.ca.c.spec$pch[all.ca.c.spec$type=="W"] <- 15
> all.ca.c.spec$pch[all.ca.c.spec$type=="C"] <- 18

> all.ca.c.spec$col <- ""
> all.ca.c.spec$col[all.ca.c.spec$type=="D"] <- "red"
> all.ca.c.spec$col[all.ca.c.spec$type=="W"] <- "blue"
> all.ca.c.spec$col[all.ca.c.spec$type=="C"] <- "purple"

# lm (polinomial 4-th gives Adjusted R-squared:  0.4661; selected)
> lm4.all.ca.c.spec <- lm(lib.spec ~ time + I(time^2) + I(time^3) + I(time^4),
   data=all.ca.c.spec)

> summary(lm4.all.ca.c.spec)
Coefficients:
              Estimate Std. Error t value Pr(>|t|)
(Intercept)  6.997e-01  2.089e-02  33.494  < 2e-16 ***
time        -4.253e-02  4.480e-03  -9.492  < 2e-16 ***
I(time^2)    1.797e-03  2.687e-04   6.687 3.14e-10 ***
I(time^3)   -2.660e-05  5.566e-06  -4.779 3.78e-06 ***
I(time^4)    1.297e-07  3.701e-08   3.504 0.000585 ***
---
Residual standard error: 0.1042 on 170 degrees of freedom
Multiple R-squared:  0.4784,Adjusted R-squared:  0.4661
F-statistic: 38.98 on 4 and 170 DF,  p-value: < 2.2e-16

# Note: lm of order lower than 4 not shown (not good fitting)
> pr.lm4.all.ca.c.spec <- as.data.frame(predict.lm(lm4.all.ca.c.spec,
   newdata=data.frame(time=c(0:80)),
  interval="prediction", level=0.95))
```

```
> pr.lm4.all.ca.c.spec$time <- c(0:80)

# Figure 3 in main text
plot(all.ca.c.spec$time, all.ca.c.spec$lib.spec, pch=all.ca.c.spec$pch, col=all.ca.c.spec$col,
xlab="Time (DAA)", ylab="Specialization")
points(seq(0, 80, by=10), tapply(all.ca.c.spec$lib.spec, all.ca.c.spec$time, mean),
   pch=10, cex=1.5)
points(pr.lm4.all.ca.c.spec$time, pr.lm4.all.ca.c.spec$fit, type="l", lwd=2)
points(pr.lm4.all.ca.c.spec$time, pr.lm4.all.ca.c.spec$lwr, type="l", lwd=2,
   lty=2, col="grey")
points(pr.lm4.all.ca.c.spec$time, pr.lm4.all.ca.c.spec$upr, type="l", lwd=2,
   lty=2, col="grey")
legend(5, 1.075,
legend=c("Domesticated", "Wild", "Cross", "Mean per time"),
pch=c(16, 15, 18, 10, 0, 0), col=c("red", "blue", "purple", "black"))
legend("topright",
legend=c("Model (Polynomial 4 dg.)", "R^2 = 0.47", "95% CI for predictions."),
   lwd=c(2,0,2), lty=c(1,1,2), col=c("black", "white", "grey"))
---------------------------------------------------------------------------------------
```

SI.5.3. **Computations for “Analyses by genotype”.**

```
---------------------------------------------------------------------------------------
# Box 3. Computations for analyses by genotype.
# Create a data.frame which will have only data of fruits adding by times of development.
> ca.fru <- ca.c[, c(1:179)[ca.c.def$fruit==T]]
> ca.fru.def <- ca.c.def[c(1:179)[ca.c.def$fruit==T], 1:5]
# Add libraries per source (in variable acc)
> ca.acc <- sum.by.var(x=ca.fru, x.def=ca.fru.def, var="acc")
> sum(ca.acc)/1e6 # Total reads in millions
[1] 2957.498
> round(apply(ca.acc,2,sum)/1e6) # Reads in millions per acc
 CM  CO  CQ  CW  QC  QU  ST  ZU  AS  JE  SR  SY
250 232 279 294 236 221 205 226 296 274 224 221
> summary(apply(ca.acc,2,sum)/1e6) # Summay reads in millions per acc
   Min. 1st Qu.  Median    Mean 3rd Qu.    Max.
  205.2   223.2   234.3   246.5   275.1   296.0
# Estimate all parameters:
> ca.acc.est <- est.all(ca.acc)
### Summarize by objects
> summ.ca.acc.est <- cbind(ca.acc.est$diversity[,c(1,2,4:8)], ca.acc.est$spec[,5:6])
# Add "type" to summ.ca.acc.est
summ.ca.acc.est$type <- ""
for(i in 1:12){
summ.ca.acc.est$type[i] <- acc$acc.type[summ.ca.acc.est$lib.name[i]==acc$acc.key]
}
> summ.ca.acc.est <- summ.ca.acc.est[,c(1,10,2:9)] # Rearrange

> ca.acc.locus <- ca.acc.est$locus
# Filter: only pi>0
> nrow(ca.acc.locus)
[1] 35883
```

```
> nrow(ca.acc.locus[ca.acc.locus$pi>0,])
[1] 33007
> ca.acc.locus <- ca.acc.locus[ca.acc.locus$pi>0,]
# Complete "ca.acc.locus" with frequencies.
> temp <- as.data.frame(ca.acc.est$freq)
> temp.2 <- apply(temp, 1, sum)
> temp <- temp[temp.2>0,]
> ca.acc.locus <- cbind(ca.acc.locus, temp)

# Are the number of genes estimated by type very different?
> summary(aov(non.0 ~ type, data= summ.ca.acc.est))
            Df Sum Sq Mean Sq F value Pr(>F)
type         2 601022  300511   3.273 0.0854 .
Residuals    9 826212   91801
# Conclusion: No, there is not a strong difference.

# Now parameters related to diversity: "H", "rel.H", "enl", "rel.enl"
> summary(aov(H ~ type, data= summ.ca.acc.est))
            Df  Sum Sq Mean Sq F value Pr(>F)
type         2 0.04114 0.02057   1.541  0.266
Residuals    9 0.12014 0.01335
> summary(aov(rel.H ~ type, data= summ.ca.acc.est))
            Df    Sum Sq   Mean Sq F value Pr(>F)
type         2 0.0002087 1.044e-04   1.857  0.211
Residuals    9 0.0005057 5.619e-05
> summary(aov(enl ~ type, data= summ.ca.acc.est))
            Df  Sum Sq Mean Sq F value Pr(>F)
type         2  576117  288058    1.39  0.298
Residuals    9 1864629  207181
> summary(aov(rel.enl ~ type, data= summ.ca.acc.est))
            Df    Sum Sq   Mean Sq F value Pr(>F)
type         2 0.0007852 0.0003926   1.769  0.225
Residuals    9 0.0019971 0.0002219
# Conclusion: Variations in diversity are not significant between types.
# However, that could be due to the inclusion of the C class, which has one outlier.
# Redo the analyses excluding that class:
> temp <- summ.ca.acc.est[summ.ca.acc.est$type != "C",]
# Use t-test (because we have only 2 classes: D and W)
> t.test(enl ~ type, data=temp)
data:  enl by type
t = -0.22532, df = 7.72, p-value = 0.8276
alternative hypothesis: true difference in means between group D and group W is not equal to 0
95 percent confidence interval:
 -522.6912  430.1726
sample estimates:
mean in group D mean in group W
       6036.407        6082.666
# Conclusion: There is no significant difference in diversity between sources.

# Specialization
> summary(aov(lib.spec ~ type, data= summ.ca.acc.est))
            Df    Sum Sq   Mean Sq F value  Pr(>F)
```

```
type         2 0.0015784 0.0007892   8.431 0.00865 **
Residuals    9 0.0008424 0.0000936
# This is significant and also
> summary(aov(rel.lib.spec ~ type, data= summ.ca.acc.est))
            Df    Sum Sq   Mean Sq F value  Pr(>F)
type         2 1.228e-04 6.141e-05   8.431 0.00865 **
Residuals    9 6.555e-05 7.280e-06
# Will keep only
> aov.lib.spec.type <- aov(lib.spec ~ type, data= summ.ca.acc.est)

# I will center the analyses in enl (diversity) and lib.spec (specialization); thus:
> lib.spec.means <- model.tables(aov.lib.spec.type, type="means")
> lib.spec.means # p-value: 0.00865
Tables of means
Grand mean
0.09538486
 type
         C       D      W
    0.1046 0.08397 0.1079
rep 2.0000 6.00000 4.0000
> aov.enl.type <- aov(enl ~ type, data= summ.ca.acc.est)
> summary(aov.enl.type)
            Df  Sum Sq Mean Sq F value Pr(>F)
type         2  576117  288058    1.39  0.298
Residuals    9 1864629  207181
> enl.means <- model.tables(aov.enl.type, type="means")
> enl.means # p-value: 0.298
Tables of means
Grand mean
5957.359
 type
       C    D    W
    5470 6036 6083
rep    2    6    4

# Plot (some details to obtain pch and col not shown)
# This is Figure 4 in MAIN TEXT
plot(summ.ca.acc.est$enl, summ.ca.acc.est$lib.spec,
pch=summ.ca.acc.est$pch, col=summ.ca.acc.est$col,
xlab="Diversity (enl)",
ylab="Specialization (lib.spec)", cex=1.5, xlim=c(4800,6500))
# Text with accession names
text(summ.ca.acc.est$enl, summ.ca.acc.est$lib.spec,
   labels=summ.ca.acc.est$lib.name, font=2, cex=0.8, pos=4)
# General means (pch=13)
points(enl.means$tables[[1]], lib.spec.means$tables[[1]], pch=13, cex=1.5)
# Grey lines in the general means
abline(h=lib.spec.means$tables[[1]], lty=2, col="grey")
abline(v=enl.means$tables[[1]], lty=2, col="grey")
# Means per type; order "C", "D", "W" pch=c(22,1,2)
points(enl.means$tables[[2]], lib.spec.means$tables[[2]], pch=c(22,1,2), cex=1.5)
legend("bottomleft", bty="n",
```

```
legend=c("General means", "Domesticated", "Wild", "Cross"),
pch=c(13, 19, 17, 15),
col=c("black", "red2", "blue2", "purple2"))

# Standardize and obtain dendrogram (Figure 2 here)
> summ.ca.acc.est$s.enl <-
  ((summ.ca.acc.est$enl-mean(summ.ca.acc.est$enl)))/sd(summ.ca.acc.est$enl)
>  summ.ca.acc.est$s.lib.spec <-
  ((summ.ca.acc.est$lib.spec-mean(summ.ca.acc.est$lib.spec)))/sd(summ.ca.acc.est$lib.spec)
> hclu.div.esp <- hclust(dist(summ.ca.acc.est[,13:14]))
# Plot the dendrogram (Figure 2)
plot(hclu.div.esp, lwd=2, main="")
grid(nx=NA, ny=NULL, lty=2, col="grey")
-------------------------------------------------------------------------------------------
```

```
-------------------------------------------------------------------------------------------
# Box 4. Study of loci specificity among sources.
# Obtain the maximum expression per accession for each locus:
> ca.acc.locus$maxAcc <- apply(ca.acc.locus[, 4:15], 1, max)

# Obtain variables to point in which accession and type are maxima
> temp <- data.frame(ca.acc.locus[, 4:15]==ca.acc.locus$maxAcc)
> ca.acc.locus$acc.max <- ""
> ca.acc.locus$type.max <- ""
for(i in 1:33007){
ca.acc.locus$acc.max[i] <- names(temp)[temp[i,]==TRUE]
ca.acc.locus$type.max[i] <- acc$acc.type[acc$acc.key==ca.acc.locus$acc.max[i]]
}

# Analysis will be focus in rel.Si (relative gene specificity)
> nrow(ca.acc.locus) # Number of expressed genes by source
[1] 33007
> summary(ca.acc.locus$rel.Si)
     Min.   1st Qu.    Median      Mean   3rd Qu.      Max.
0.0002318 0.0087176 0.0426435 0.1549014 0.1757224 1.0000000
# Obviously, there is a strong positive correlation between the
# mean expression (pi) and the maximum expression per accession (maxAcc)
> cor(ca.acc.locus$pi, ca.acc.locus$maxAcc)
[1] 0.9633293

# Figure 3 (distributions of rel.Si and rel.Si by accession)
# Figure 3 A: Histogram of rel.Si
hist(ca.acc.locus$rel.Si)
abline(v=c(0.0426435, 0.1549014), lty=c(2,3), lwd=2, col="red")
legend("topright", bty="n", legend=c("Median = 0.04", "Mean = 0.16"),
   lty=c(2,3), lwd=2, col="red")
# Figure 3 B: Box plots of rel.Si by accession where maximum was reached
boxplot(rel.Si ~ acc.max, data=ca.acc.locus, outline=F)
# Plot means as asterisks
text(c(1:12), tapply(ca.acc.locus$rel.Si, ca.acc.locus$acc.max, mean),
   labels="*", col="red", cex=2)
# Put number of cases as brown figures in each distribution
```

```
text(c(1:12), rep(0.65, 12), labels=tapply(ca.acc.locus$rel.Si, ca.acc.locus$acc.max, length),
    font=2, col="brown", srt = 90)
legend("topleft", bty="n", legend=c("Mean",
   "Number of cases in brown figures."), pch=c(8,NA), col="red")

# Statistics of number of cases and mean rel.Si per accession.
> temp <- rbind(tapply(ca.acc.locus$rel.Si, ca.acc.locus$acc.max, length),
tapply(ca.acc.locus$rel.Si, ca.acc.locus$acc.max,length)/nrow(ca.acc.locus),
tapply(ca.acc.locus$rel.Si, ca.acc.locus$acc.max, mean))
> temp <- as.data.frame(t(temp))
> names(temp) <- c("Cases", "RelFreCases", "MeanRelSi")
> temp <- temp[order(temp$MeanRelSi),] # Order by MeanRelSi
> temp
   Cases RelFreCases  MeanRelSi
CW  4914  0.14887751 0.07772064
ZU  2776  0.08410337 0.11680406
CM  2203  0.06674342 0.13748665
ST  2639  0.07995274 0.14643118
JE  2712  0.08216439 0.15592012
CO  2116  0.06410761 0.15621845
QU  3379  0.10237222 0.16131794
AS  2718  0.08234617 0.17022303
QC  1762  0.05338262 0.17346461
SY  3695  0.11194595 0.20730735
SR  3314  0.10040294 0.21411896
CQ   779  0.02360105 0.22457076

# Statistics of number of cases and mean rel.Si per type.
> temp <- rbind(tapply(ca.acc.locus$rel.Si, ca.acc.locus$type.max, length),
tapply(ca.acc.locus$rel.Si, ca.acc.locus$type.max,length)/nrow(ca.acc.locus),
apply(ca.acc.locus$rel.Si, ca.acc.locus$type.max, mean))
> temp <- as.data.frame(t(temp))
names(temp) <- c("Cases", "RelFreCases", "MeanRelSi")
> temp <- temp[order(temp$MeanRelSi),] # Order by MeanRelSi
> temp
  Cases RelFreCases MeanRelSi
D 17962  0.54418760 0.1269905
W 12504  0.37882873 0.1880392
C  2541  0.07698367 0.1891323
# Summaries of ANOVAS for rel.Si group by accessions and groups.
> aov.rel.SiXacc <- aov(rel.Si ~ acc.max, data=ca.acc.locus)
> summary(aov.rel.SiXacc)
               Df Sum Sq Mean Sq F value Pr(>F)
acc.max        11   61.1   5.555   96.49 <2e-16 ***
Residuals   32995 1899.5   0.058

> aov.rel.SiXtype <- aov(rel.Si ~ type.max, data=ca.acc.locus)
> summary(aov.rel.SiXtype)
               Df Sum Sq Mean Sq F value Pr(>F)
type.max        2   30.7  15.350   262.5 <2e-16 ***
Residuals   33004 1929.9   0.058
> TukeyHSD(aov.rel.SiXtype)
```

```
  Tukey multiple comparisons of means
    95% family-wise confidence level

Fit: aov(formula = rel.Si ~ type.max, data = ca.acc.locus)

$type.max
           diff         lwr         upr     p adj
D-C -0.06214181 -0.07415366 -0.05012995 0.0000000
W-C -0.00109311 -0.01342559  0.01123937 0.9764903
W-D  0.06104870  0.05444805  0.06764935 0.0000000
-----------------------------------------------------------------------------------

-----------------------------------------------------------------------------------
# Box 5. Gene Ontology enrichment analyses of "generalist" genes.
# Select the identifiers of generalist loci, i.e., genes with less
# than 0.05 rel.Si
> generalist.ids <- as.integer(attributes(
ca.acc.locus[ca.acc.locus$rel.Si<=0.05,])$row.names)
> length(generalist.ids) # How many do we have?
[1] 17333
# Examples of some of those genes
> head(gene[is.element(gene$id, generalist.ids), 1:3])
   id         ProtId
19 19 XP_016545450.1
26 26 XP_016545534.1
27 27 XP_016545431.1
28 28 XP_016545540.1
30 30 XP_016545761.1
31 31 XP_016545770.1
                                                Prot.Desc
19                  dynamin-like protein ARC5 isoform X5
26 cell cycle checkpoint control protein RAD9A isoform X2
27          aspartic proteinase-like protein 2 isoform X1
28              phosphoglycerate mutase-like protein AT74
30                  uncharacterized protein LOC107845793
31                           tubby-like F-box protein 10

# Enrichment for Biological Process (BP)
> GO.BP.generalist <- analyze.all.GO(generalist.ids, aspect="BP", only.FDR.le=0.01)
# Enrichment for Molecular Function (MF)
> GO.MF.generalist <- analyze.all.GO(generalist.ids, aspect="MF", only.FDR.le=0.01)
# Enrichment for Cell Component (CC)
GO.CC.generalist <- analyze.all.GO(generalist.ids, aspect="CC", only.FDR.le=0.01)

# NOTES (further analyses)
# To perform manual curation of the GO enrichment analyses we programed functions
# "groupGOresults", "SelectFromGroups" and "dist2data"; object: "groupGOresults.R".
# Also, we programed the function "getGenesFromEnriched()" which allow to obtain
# the gene identifiers and descriptions of genes that were used in a GO enrichment.
# All those objects and results are available upon request.
-----------------------------------------------------------------------------------

-----------------------------------------------------------------------------------
```

```
# Box 6. Genes with rel.Si > 0.5
# Classifying all loci in discrete classes in "ca.acc.locus"
# Will make a categorical variable "rel.Si.Int" in ca.acc.locus
> temp.ls <- seq(0.1, 1, by=0.1)
> temp.li <- seq(0, 0.9, by=0.1)
> temp.I <- paste(c(1:10), paste(temp.li, temp.ls, sep="to"),sep="-")
> temp.I
 [1] "1-0to0.1"   "2-0.1to0.2" "3-0.2to0.3" "4-0.3to0.4" "5-0.4to0.5"
 [6] "6-0.5to0.6" "7-0.6to0.7" "8-0.7to0.8" "9-0.8to0.9" "10-0.9to1"

> ca.acc.locus$rel.Si.Int <- ""
 for(i in 1:10){
temp <- (ca.acc.locus$rel.Si>=temp.li[i])&(ca.acc.locus$rel.Si<=temp.ls[i])
ca.acc.locus$rel.Si.Int[c(1:33007)[temp==TRUE]] <- temp.I[i]
}

> table(ca.acc.locus$rel.Si.Int)
            1-0to0.1  10-0.9to1 2-0.1to0.2 3-0.2to0.3 4-0.3to0.4
       638      21663        541       3713       1907       1255
5-0.4to0.5 6-0.5to0.6 7-0.6to0.7 8-0.7to0.8 9-0.8to0.9
      1069        825        421        832        143
> summary(ca.acc.locus$rel.Si[ca.acc.locus$rel.Si.Int==""])
   Min. 1st Qu.  Median    Mean 3rd Qu.    Max.
      1       1       1       1       1       1
> ca.acc.locus$rel.Si.Int[ca.acc.locus$rel.Si.Int==""] <- "10-0.9to1"
# See if we have the correct number of cases:
> sum(table(ca.acc.locus$rel.Si.Int))
[1] 33007
> temp <- table(ca.acc.locus$type.max, ca.acc.locus$rel.Si.Int)
> temp <- temp[,c(1,3:10,2)] # reorder columns
> temp <- temp[c(2,3,1),] # Reorder rows
> temp
    1-0to0.1 2-0.1to0.2 3-0.2to0.3 4-0.3to0.4 5-0.4to0.5 6-0.5to0.6
  D    13072       1608        792        525        455        351
  W     7106       1747        936        616        536        398
  C     1485        358        179        114         78         76

    7-0.6to0.7 8-0.7to0.8 9-0.8to0.9 10-0.9to1
  D        168        358         63       570
  W        229        394         74       468
  C         24         80          6       141

> n.gen.by.relSi <- temp # Put the table
> n.gen.by.relSi[,6:10]
    6-0.5to0.6 7-0.6to0.7 8-0.7to0.8 9-0.8to0.9 10-0.9to1
  D        351        168        358         63       570
  W        398        229        394         74       468
  C         76         24         80          6       141

# Add totals to n.gen.by.relSi
> n.gen.by.relSi <- rbind(n.gen.by.relSi, apply(n.gen.by.relSi,2,sum))
> n.gen.by.relSi <- cbind(n.gen.by.relSi, apply(n.gen.by.relSi,1,sum))
```

```
> attributes(n.gen.by.relSi)$dimnames[[1]][4] <- "Total"
> attributes(n.gen.by.relSi)$dimnames[[2]][11] <- "Total"
> n.gen.by.relSi
      1-0to0.1 2-0.1to0.2 3-0.2to0.3 4-0.3to0.4 5-0.4to0.5 6-0.5to0.6
D        13072       1608        792        525        455        351
W         7106       1747        936        616        536        398
C         1485        358        179        114         78         76
Total    21663       3713       1907       1255       1069        825
      7-0.6to0.7 8-0.7to0.8 9-0.8to0.9 10-0.9to1 Total
D            168        358         63       570 17962
W            229        394         74       468 12504
C             24         80          6       141  2541
Total        421        832        143      1179 33007

> round(100*n.gen.by.relSi/33007, 2) # In % (rounded)
      1-0to0.1 2-0.1to0.2 3-0.2to0.3 4-0.3to0.4 5-0.4to0.5 6-0.5to0.6
D        39.60       4.87       2.40       1.59       1.38       1.06
W        21.53       5.29       2.84       1.87       1.62       1.21
C         4.50       1.08       0.54       0.35       0.24       0.23
Total    65.63      11.25       5.78       3.80       3.24       2.50
      7-0.6to0.7 8-0.7to0.8 9-0.8to0.9 10-0.9to1  Total
D           0.51       1.08       0.19      1.73  54.42
W           0.69       1.19       0.22      1.42  37.88
C           0.07       0.24       0.02      0.43   7.70
Total       1.28       2.52       0.43      3.57 100.00

# Make Figure 5 (in main text)
temp <- barplot(n.gen.by.relSi[1:3,6:10],  col=c("red", "blue", "violet"), ylim=c(0,1250),
   xlab="Interval of values for rel.Si", ylab="Frequency of loci")
legend("topleft", bty="n", legend=c("C", "W", "D"), fill=c("violet", "blue", "red"))
# Total per bar (interval)
text(temp, n.gen.by.relSi[4,6:10]+20, labels=n.gen.by.relSi[4,6:10], font=2)
# Number of domesticated
text(temp, n.gen.by.relSi[1,6:10]/2, labels=n.gen.by.relSi[1,6:10], font=2, col="white")
# Number of wild
text(temp, n.gen.by.relSi[1,6:10] +  n.gen.by.relSi[2,6:10]/2, labels=n.gen.by.relSi[2,6:10],
  font=2, col="white")

# We are going to do two GO analyses of specific genes:
# First: With the most specific independently of type:
> ids.HiSi <- as.integer(attributes(ca.acc.locus
   [ca.acc.locus$rel.Si.Int=="10-0.9to1",])$row.names)
> length(ids.HiSi) # Must be 1179
[1] 1179
# In D with rel.Si > 0.7 we have: 358+63+570=991
ids.HiSiD <-
as.integer(attributes(ca.acc.locus[(ca.acc.locus$type.max=="D")&
((ca.acc.locus$rel.Si.Int=="8-0.7to0.8")|(ca.acc.locus$rel.Si.Int=="9-0.8to0.9")|
     (ca.acc.locus$rel.Si.Int=="10-0.9to1")),])$row.names)
> length(ids.HiSiD)
[1] 991
```

```
# In W with rel.Si > 0.7 we have: 394+74+468 = 936
ids.HiSiW <-
as.integer(attributes(ca.acc.locus[(ca.acc.locus$type.max=="W")&((ca.acc.locus$rel.Si.In
  t=="8-0.7to0.8")|(ca.acc.locus$rel.Si.Int=="9-0.8to0.9")|
  (ca.acc.locus$rel.Si.Int=="10-0.9to1")),])$row.names)
length(ids.HiSiW)
[1] 936

# Note: Not relevant results were obtained for ids.HiSi
# Continuing analyses with sets ids.HiSiD and ids.HiSiW

# ids.HiSiD (991 genes)
GO.BP.HiSiD <- analyze.all.GO(ids.HiSiD, aspect="BP", only.FDR.le=0.01)
GO.MF.HiSiD <- analyze.all.GO(ids.HiSiD, aspect="MF", only.FDR.le=0.01)
GO.CC.HiSiD <- analyze.all.GO(ids.HiSiD, aspect="CC", only.FDR.le=0.01)
# ids.HiSiW (936 genes)
GO.BP.HiSiW <- analyze.all.GO(ids.HiSiW, aspect="BP", only.FDR.le=0.01)
GO.MF.HiSiW <- analyze.all.GO(ids.HiSiW, aspect="MF", only.FDR.le=0.01)
GO.CC.HiSiW <- analyze.all.GO(ids.HiSiW, aspect="CC", only.FDR.le=0.01)

# Contrasting BP between W and D:
> GO.BP.HiSiW$desc
[1] "proteolysis"               "protein metabolic process"
[3] "metabolic process"
> GO.BP.HiSiD$desc
[1] "proteolysis"
[2] "photosynthesis"
[3] "single-organism process"
[4] "multi-organism process"
[5] "protein metabolic process"
[6] "single-organism cellular process"
> setdiff(GO.BP.HiSiD$desc, GO.BP.HiSiW$desc) # In D but not in W
[1] "photosynthesis"
[2] "single-organism process"
[3] "multi-organism process"
[4] "single-organism cellular process"

# Contrasting MF between W and D:
> GO.MF.HiSiW$desc
[1] "cysteine-type peptidase activity"
[2] "peptidase activity, acting on L-amino acid peptides"
[3] "peptidase activity"
> GO.MF.HiSiD$desc
[1] "cysteine-type peptidase activity"
[2] "peptidase activity, acting on L-amino acid peptides"
[3] "peptidase activity"
[4] "protein binding"
[5] "binding"
> setdiff(GO.MF.HiSiD$desc, GO.MF.HiSiW$desc) # In D but not in W
[1] "protein binding" "binding"

# Conclusions:
```

```
# 1 - Genes with high specificity (in D or W) are differentially enriched
# in the BP "photosynthesis" in D but NOT in W.
# 2 - Genes with high specificity (in D or W) are differentially enriched
# in the MF "binding" in D but NOT in W.
# However, it will be better (more discriminative) to make an analysis
# per type of accession ("D" or "W") without taking into account time.
--------------------------------------------------------------------------------
```

```
--------------------------------------------------------------------------------
# Box 7. Grouping expression in fruit by type (D and W sources).
# Define the target groups:
> temp.f <- ca.c[,attributes(ca.c.def[ca.c.def$fruit==T,])$row.names]
> temp.f.def <- ca.c.def[ca.c.def$fruit==T,]
# Obtain the desired data.frame of counts
> temp.f2 <- sum.by.var(x=temp.f, x.def=temp.f.def, var="type")
# But we want to analyze only D and W (but not C), so:
> ca.fruit.DW <- temp.f2[,1:2] # Only D and W
# Filter (only expressed genes)
> ca.fruit.DW <- ca.fruit.DW[c(1:35883)[temp>0],]
# Estimate all transcriptome parameters:
> ca.fruit.DW.all <- est.all(ca.fruit.DW)

> names(ca.fruit.DW.all)
[1] "diversity" "spec"      "locus"     "freq"
> ca.fruit.DW.all[[1]]
  lib.name     n.tags lib.length non.0        H     rel.H      enl
1        D 1544124339      32860 31979 12.64554 0.8450170 6407.484
2        W  897738654      32860 31729 12.61043 0.8433084 6253.400
    rel.enl
1 0.2003654
2 0.1970878
> ca.fruit.DW.all[[2]]
  lib.name     n.tags lib.length non.0   lib.spec rel.lib.spec
D        D 1544124339      32860 31979 0.02962672   0.02962672
W        W  897738654      32860 31729 0.04137311   0.04137311

# Prepare the results to be presented as Table 7.
> table7 <- cbind(ca.fruit.DW.all[[1]][,c(1,2,4,7,8)], ca.fruit.DW.all[[2]][,5:6])
> table7
  lib.name     n.tags non.0      enl   rel.enl   lib.spec rel.lib.spec
D        D 1544124339 31979 6407.484 0.2003654 0.02962672   0.02962672
W        W  897738654 31729 6253.400 0.1970878 0.04137311   0.04137311
> 0.04137311/0.02962672 # rel.lib.spec W / D
[1] 1.39648
> table7$n.tags <- round(table7$n.tags/1e6) # Express in millions
> table7$enl <- round(table7$enl) # Rounding
> table7[,5:7] <- round(table7[,5:7], 5) # Rounding
> table7
  lib.name n.tags non.0  enl rel.enl lib.spec rel.lib.spec
D        D   1544 31979 6407 0.20037  0.02963      0.02963
W        W    898 31729 6253 0.19709  0.04137      0.04137
# (presented as Table 7 with modified labels)
```

```
# We are going to center the analysis in the loci, so
> ca.fruit.DW.loci <- ca.fruit.DW.all[[3]]
# Let's add the frequencies of expression
> ca.fruit.DW.loci <- cbind(ca.fruit.DW.loci, as.data.frame(ca.fruit.DW.all[[4]]))
# Now let's add two logical variables:
> ca.fruit.DW.loci$FE <- ca.fruit.DW.loci$rel.Si==1 # Fully Specific
> ca.fruit.DW.loci$MoreInD <- ca.fruit.DW.loci$D > ca.fruit.DW.loci$W
# Rows are Fully specific and columns are larger in D and W
> table(ca.fruit.DW.loci$FE, ca.fruit.DW.loci$MoreInD)
        FALSE  TRUE
  FALSE 16192 14656 <- Not fully specific
  TRUE    881  1131 <- Fully specific
#       W     D
# Note: The row attributes go from c(1:32860) now we will analyze
# the sets of fully specific genes per group:
> temp.W <- c(1:32860)[(ca.fruit.DW.loci$FE==TRUE)&(ca.fruit.DW.loci$MoreInD==FALSE)]
> ids.FE.W <- as.integer(attributes(ca.fruit.DW.loci)$row.names[temp.W])

> temp.D <- c(1:32860)[(ca.fruit.DW.loci$FE==TRUE)&(ca.fruit.DW.loci$MoreInD==TRUE)]
> ids.FE.D <- as.integer(attributes(ca.fruit.DW.loci)$row.names[temp.D])

# Summary of statistics for the loci.
> summary(ca.fruit.DW.loci)
       pi                   Si                 rel.Si
 Min.   :0.000e+00   Min.   :0.000000   Min.   :0.000000
 1st Qu.:1.200e-07   1st Qu.:0.002906   1st Qu.:0.002906
 Median :4.053e-06   Median :0.019297   Median :0.019297
 Mean   :3.043e-05   Mean   :0.143311   Mean   :0.143311
 3rd Qu.:2.540e-05   3rd Qu.:0.124242   3rd Qu.:0.124242
 Max.   :7.967e-03   Max.   :1.000000   Max.   :1.000000
       D                    W                  FE
 Min.   :0.000e+00   Min.   :0.000e+00   Mode :logical
 1st Qu.:8.700e-08   1st Qu.:1.140e-07   FALSE:30848
 Median :3.752e-06   Median :4.118e-06   TRUE :2012
 Mean   :3.043e-05   Mean   :3.043e-05
 3rd Qu.:2.506e-05   3rd Qu.:2.535e-05
 Max.   :1.005e-02   Max.   :8.771e-03
  MoreInD
 Mode :logical
 FALSE:17073
 TRUE :15787

# See the SEPs of genes exclusively expressed in D and W.
# Note: Not all exclusive genes have a SEP
> ids.FE.D.SEPS <- intersect(ids.FE.D, SEP$id[SEP$acc.type=="D"])
> length(ids.FE.D.SEPS)
[1] 191
> SEPs.FE.D <- get.SEP(ids.FE.D.SEPS, acc.type="D")
> nrow(SEPs.FE.D)
[1] 323
> table(SEPs.FE.D$acc.key)
```

```
AS CM CW JE ST ZU
59 53 46 57 57 51
> SEPs.plot(SEPs.FE.D) # Decreasing!

> length(ids.FE.W)
[1] 881
> ids.FE.W.SEPS <- intersect(ids.FE.W, SEP$id[SEP$acc.type=="W"])
> length(ids.FE.W.SEPS)
[1] 157
> SEPs.FE.W <- get.SEP(ids.FE.W.SEPS, acc.type="W")
> nrow(SEPs.FE.W)
[1] 232
> table(SEPs.FE.W$acc.key)
CO QU SR SY
39 50 70 73

# Summaries of the SEPs of FE genes:
> SEP.summary(SEPs.FE.D, SEPs.FE.W)
$general
    n.rows n.ids n.acc n.type n.mod mean.TimeMaxExp LL.TimeMaxExp
g.1    323   191     6      1   110        23.37461      21.23843
g.2    232   157     4      1    89        29.09483      26.50898
    UL.TimeMaxExp
g.1      25.51079
g.2      31.68068

$time.means
          m.T0       m.T10       m.T20        m.T30       m.T40
g.1 0.40237841  0.01331454 -0.02020514 -0.002898741 -0.08431179
g.2 0.04549411 -0.02460190  0.03824053  0.029506086  0.03609937
         m.T50        m.T60
g.1 -0.1519933 -0.156284001
g.2 -0.1321570  0.007418832

$LL.time.means
       LL.m.T0    LL.m.T10    LL.m.T20    LL.m.T30    LL.m.T40
g.1  0.2752031 -0.07714604 -0.09361025 -0.08420659 -0.15907730
g.2 -0.1088448 -0.12285148 -0.06121050 -0.07089320 -0.06591382
      LL.m.T50   LL.m.T60
g.1 -0.2269484 -0.2557303
g.2 -0.2162038 -0.1175173

$UL.time.means
      UL.m.T0   UL.m.T10   UL.m.T20  UL.m.T30     UL.m.T40    UL.m.T50
g.1 0.5295538 0.10377512 0.05319998 0.0784091 -0.009546291 -0.07703814
g.2 0.1998330 0.07364768 0.13769156 0.1299054  0.138112563 -0.04811029
       UL.m.T60
g.1 -0.05683772
g.2  0.13235496

# Presented in main text as Figure 6.
SEPs.plot(list(SEPs.FE.D, SEPs.FE.W), colors=c("red", "blue"))
```

```
legend("topright", bty="n",
 legend=c("D, 191 genes in 6 acc.", "W, 157 genes in 4 acc."),
 col=c("red", "blue"), pch=1, lty=1, lwd=2)

# Now, let's detect the genes with higher expression at 0 DAA in D
> head(SEPs.FE.D[order(SEPs.FE.D$seT0, decreasing=T), c(1,2,4,6)], 2)
       id acc.key  model     seT0
2164 3056      AS DSSSSS 2.267787
2790 3919      AS DSSSSS 2.267787
> nrow(SEPs.FE.D[SEPs.FE.D$seT0>2.26,])
[1] 60
> length(unique(SEPs.FE.D$id[SEPs.FE.D$seT0>2.26]))
[1] 43
> table(SEPs.FE.D$acc.key[SEPs.FE.D$seT0>2.26])
AS CM CW JE ST ZU
12 16  9 12  6  5
# Are there TF in that group?
> gene[(is.element(gene$id, unique(SEPs.FE.D$id[SEPs.FE.D$seT0>2.26])))&(gene$isTF==TRUE),]
         id         ProtId                     Prot.Desc isTF
17631 17631 XP_016542429.1 transcription factor AS1-like TRUE

# At NCBI:
# https://www.ncbi.nlm.nih.gov/search/all/?term=XP_016542429.1

# Now the same but for W
> head(SEPs.FE.W[order(SEPs.FE.W$seT0, decreasing=T), c(1,2,4,6)], 2)
         id acc.key  model     seT0
52964  1619      CO DSSSSS 2.267787
68715 23578      CO DSSSSS 2.267787
> nrow(SEPs.FE.W[SEPs.FE.W$seT0>2.26,])
[1] 34
> length(unique(SEPs.FE.W$id[SEPs.FE.W$seT0>2.26]))
[1] 27
> table(SEPs.FE.W$acc.key[SEPs.FE.W$seT0>2.26])
CO QU SR SY
 3  3 15 13
# Are there TF in that group?
> gene[(is.element(gene$id, unique(SEPs.FE.W$id[SEPs.FE.W$seT0>2.26])))&(gene$isTF==TRUE),]
[1] id        ProtId    Prot.Desc isTF
<0 rows> (or 0-length row.names)

# Without restricting by maximum expression at 0 DAA
 # Are other TF in the genes exclusively expressed in D?
> gene[(!is.na(gene$Prot.Desc))&(is.element(gene$id, ids.FE.D))&(gene$isTF==TRUE), ]
         id         ProtId
5723   5723 XP_016540811.1
8881   8881 XP_016567970.1
17631 17631 XP_016542429.1
17753 17753 XP_016544751.1
29354 29354 XP_016581393.1
                                           Prot.Desc isTF
5723                     transcription factor TGA6-like TRUE
```

```
8881       nuclear transcription factor Y subunit B-5-like TRUE
17631                        transcription factor AS1-like TRUE
17753 ethylene-responsive transcription factor ERF109-like TRUE
29354             scarecrow-like transcription factor PAT1 TRUE

# And what about in W?
> gene[(!is.na(gene$Prot.Desc))&(is.element(gene$id, ids.FE.W))&(gene$isTF==TRUE), ]
         id         ProtId
2737   2737 XP_016577487.1
10536 10536 XP_016582130.1
                                                                 Prot.Desc
2737  B3 domain-containing transcription factor ABI3-like isoform X3
10536   zinc finger BED domain-containing protein RICESLEEPER 2-like
      isTF
2737  TRUE
10536 TRUE

### Formatting those results for Table 8 in main text:
> temp.D <- gene[(!is.na(gene$Prot.Desc))&(is.element(gene$id, ids.FE.D))&
   (gene$isTF==TRUE), 1:3]
> temp.W <- gene[(!is.na(gene$Prot.Desc))&(is.element(gene$id, ids.FE.W))&
   (gene$isTF==TRUE), 1:3]
> temp.D$EEin <- "D"
> temp.W$EEin <- "W"
> table8 <- rbind(temp.D, temp.W)
> table8 <- table8[,c(4,1:3)]

# Now the GO analyses for those genes [Will be less stringent]
# ids.FE.W (881 genes)
GO.BP.FE.W <- analyze.all.GO(ids.FE.W, aspect="BP", only.FDR.le=0.1)
GO.MF.FE.W <- analyze.all.GO(ids.FE.W, aspect="MF", only.FDR.le=0.1)
GO.CC.FE.W <- analyze.all.GO(ids.FE.W, aspect="CC", only.FDR.le=0.1)

# ids.FE.D (1131 genes)
GO.BP.FE.D <- analyze.all.GO(ids.FE.D, aspect="BP", only.FDR.le=0.1)
GO.MF.FE.D <- analyze.all.GO(ids.FE.D, aspect="MF", only.FDR.le=0.1)
GO.CC.FE.D <- analyze.all.GO(ids.FE.D, aspect="CC", only.FDR.le=0.1)
-------------------------------------------------------------------------------------
```

SI.5.4. **Computations for “Analyses by time of development”.**

```
-------------------------------------------------------------------------------------
# Box 8.  Analysis by time.
# Select only fruit columns:
> temp.f <- ca.c[,attributes(ca.c.def[ca.c.def$fruit==T,])$row.names]
> dim(temp.f)
[1] 35883   175
> temp.f.def <- ca.c.def[ca.c.def$fruit==T,]
# Add columns by time
> temp.f2 <- sum.by.var(x=temp.f, x.def=temp.f.def, var="time")
> dim(temp.f2)
[1] 35883     9
```

```
# Rename adding "T.":
> names(temp.f2) <- paste("T.", names(temp.f2), sep="")
> ca.time <- temp.f2
> temp <- apply(ca.time, 1, sum) # Sum by locus
> length(temp)
[1] 35883
# Filter: Only locus expressed.
> ca.time <- ca.time[c(1:35883)[temp>0],]
> dim(ca.time)
[1] 33007     9
# Obtain all results:
> ca.time.all <- est.all(ca.time)
> names(ca.time.all) # Names of the list:
[1] "diversity" "spec"      "locus"     "freq"
> ca.time.all[[1]] # Diversity
  lib.name    n.tags lib.length non.0        H     rel.H      enl
1      T.0 405534001      33007 30381 12.73224 0.8550360 6804.344
2     T.10 367145620      33007 29834 12.63455 0.8499718 6358.853
3     T.20 404968232      33007 30321 12.60495 0.8466497 6229.697
4     T.30 398113619      33007 30263 12.40242 0.8332008 5413.767
5     T.40 436825080      33007 29994 12.07551 0.8119415 4316.085
6     T.50 409883220      33007 29594 12.06441 0.8122530 4283.003
7     T.60 391946849      33007 29384 12.02505 0.8101639 4167.755
8     T.70 109902575      33007 25716 11.91501 0.8132901 3861.667
9     T.80  33178857      33007 22112 11.43840 0.7925424 2775.249
    rel.enl
1 0.2239671
2 0.2131412
3 0.2054582
4 0.1788906
5 0.1438983
6 0.1447254
7 0.1418376
8 0.1501659
9 0.1255087
> ca.time.all[[2]] # Specialization
     lib.name    n.tags lib.length non.0  lib.spec rel.lib.spec
T.0       T.0 405534001      33007 30381 0.6065276   0.19133816
T.10     T.10 367145620      33007 29834 0.3123154   0.09852453
T.20     T.20 404968232      33007 30321 0.2630982   0.08299824
T.30     T.30 398113619      33007 30263 0.2927829   0.09236272
T.40     T.40 436825080      33007 29994 0.3373555   0.10642380
T.50     T.50 409883220      33007 29594 0.3541789   0.11173100
T.60     T.60 391946849      33007 29384 0.3389338   0.10692171
T.70     T.70 109902575      33007 25716 0.2815617   0.08882283
T.80     T.80  33178857      33007 22112 0.3382167   0.10669550

# Prepare the results to be presented as Table 10 in main text.
> table10 <- cbind(ca.time.all[[1]][,c(1,2,4,7,8)], ca.time.all[[2]][,5:6])
> table10$n.tags <- round(table10$n.tags/1e6) # Express in millions
> table10$enl <- round(table10$enl) # Rounding
> table10[,5:7] <- round(table10[,5:7], 5) # Rounding
```

```
> table10
     lib.name n.tags non.0  enl rel.enl lib.spec rel.lib.spec
T.0       T.0    406 30381 6804 0.22397  0.60653      0.19134
T.10     T.10    367 29834 6359 0.21314  0.31232      0.09852
T.20     T.20    405 30321 6230 0.20546  0.26310      0.08300
T.30     T.30    398 30263 5414 0.17889  0.29278      0.09236
T.40     T.40    437 29994 4316 0.14390  0.33736      0.10642
T.50     T.50    410 29594 4283 0.14473  0.35418      0.11173
T.60     T.60    392 29384 4168 0.14184  0.33893      0.10692
T.70     T.70    110 25716 3862 0.15017  0.28156      0.08882
T.80     T.80     33 22112 2775 0.12551  0.33822      0.10670

# Make plot Diversity (enl) by relative specialization (rel.lib.spec)
# (presented as Figure 6)
plot(table10$enl, table10$rel.lib.spec, pch=c(1:9), cex=1.5,
xlab="Diversity (enl)", ylab="Specialization (rel.lib.spec)")
points(mean(table10$enl), mean(table10$rel.lib.spec), pch=19, cex=1.5, col="red")
abline(v=mean(table10$enl), col="red")
abline(h=mean(table10$rel.lib.spec), col="red")
legend("topleft", bty="n", pch=c(1:9, 19), legend=c(table10$lib.name, "Mean"),
   text.font=2, col=c(rep("black", 9), "red"))
segments(x0=table10$enl[1:8], y0=table10$rel.lib.spec[1:8],
   x1=table10$enl[2:9], y1=table10$rel.lib.spec[2:9], lty=2, col="grey", lwd=2)

# Linear model enl X time
> t.time <- seq(0, 80, by=10)
> lm.enlXt.time <- lm(table10$enl ~ t.time)
> summary(lm.enlXt.time)
Coefficients:
            Estimate Std. Error t value Pr(>|t|)
(Intercept) 6836.467    201.546   33.92 5.02e-09 ***
t.time       -48.103      4.233  -11.36 9.16e-06 ***
Residual standard error: 327.9 on 7 degrees of freedom
Multiple R-squared:  0.9486,Adjusted R-squared:  0.9412
F-statistic: 129.1 on 1 and 7 DF,  p-value: 9.161e-06

# Make plot Diversity (enl) by time in DAA
# (presented as Figure 7)
plot(seq(0, 80, by=10), table10$enl, type="b", lwd=2,
xlab="Time (DAA)", ylab="Diversity (enl)")
grid()
abline(lm.enlXt.time, lty=2, lwd=2, col="red")
legend("topright", bty="n", box.col="white", col=c("black", "red"),
legend=c("Estimates", "Model: enl = 6836 - 48 Time",
"Adjusted R-squared:  0.94"),
pch=c(1, NA, NA), lty=c(1, 2, NA), lwd=2)

# Make plot Spec (rel.lib.spec) by time in DAA
# (presented as Figure 8)
plot(seq(0, 80, by=10), table10$rel.lib.spec, type="b", lwd=2,
xlab="Time (DAA)", ylab="Specialization (rel.lib.spec)")
```

```
grid()
# For section "Highly specific genes per time"
# Will consider as highly specific cases rel.Si > 0.9
# Will make a new variable "HiSin"
ca.time.all.loc$HiSin <- "No"
ca.time.all.loc$HiSin[(ca.time.all.loc$rel.Si>0.9)&(ca.time.all.loc$MaxIn=="T.0")] <- "T.0"
# ... and so on for the other times:
ca.time.all.loc$HiSin[(ca.time.all.loc$rel.Si>0.9)&(ca.time.all.loc$MaxIn=="T.80")] <- "T.80"

> temp<-table(ca.time.all.loc$HiSin)
> sum(temp[2:10])
[1] 1898
> temp[2:10]
 T.0 T.10 T.20 T.30 T.40 T.50 T.60 T.70 T.80
 986  117  205  149  150  100  143   26   22

# Make a boxplot (Presented as Figure 9):
boxplot(rel.Si ~ MaxIn, data=ca.time.all.loc, outline=F, notch=T,
xlab="Time (DAA)", ylab="Locus specificity (rel.Si)")
points(c(1:9), unlist(tapply(ca.time.all.loc$rel.Si, ca.time.all.loc$MaxIn, mean)),
   pch=8, col="red", cex=1.5)
# Put the rounded percentage of cases
text(c(1:9), rep(0.9, 9), labels=
round(100*table(ca.time.all.loc$MaxIn)/nrow(ca.time.all.loc),1),
col="brown", cex=1, font=2)
legend("topright", bty="n", legend="Mean", pch=8, col="red")
text(0.4, 0.9, labels="%=", col="brown", cex=1, font=2)
text(c(1:9), rep(0.95, 9), labels=temp[2:10], font=2, col="darkblue")
text(0.4, 0.95, labels="HS:", col="darkblue", cex=1, font=2)

# Now will add other variable to group get the genes with HS
# per development stage, var: HStage Flower, Growing, Maturity:
> ca.time.all.loc$HStage <- ""
> ca.time.all.loc$HStage[ca.time.all.loc$HiSin=="T.0"] <- "Flower"
# T.10, T.20, T.40, T.40: "Growing"
> ca.time.all.loc$HStage[(ca.time.all.loc$HiSin=="T.10")|
   (ca.time.all.loc$HiSin=="T.20")|(ca.time.all.loc$HiSin=="T.30")|
   (ca.time.all.loc$HiSin=="T.40")] <- "Growing"
# T.50, T.60, T.70, T.80: "Maturity"
> ca.time.all.loc$HStage[(ca.time.all.loc$HiSin=="T.50")|
   (ca.time.all.loc$HiSin=="T.60")|(ca.time.all.loc$HiSin=="T.70")|
   (ca.time.all.loc$HiSin=="T.80")] <- "Maturity"
> table(ca.time.all.loc$HStage)

           Flower  Growing Maturity
   31109      986      621      291

idsHS.Flower <-
   as.integer(attributes(ca.time.all.loc[ca.time.all.loc$HStage=="Flower",])$row.names)
idsHS.Growing <-
   as.integer(attributes(ca.time.all.loc[ca.time.all.loc$HStage=="Growing",])$row.names)
idsHS.Maturity <-
```

```
   as.integer(attributes(ca.time.all.loc[ca.time.all.loc$HStage=="Maturity",])$row.names)

# GO analyses with those ids:
GO.BP.HS.Flower <- analyze.all.GO(idsHS.Flower, aspect="BP", only.FDR.le=0.1)
GO.MF.HS.Flower <- analyze.all.GO(idsHS.Flower, aspect="MF", only.FDR.le=0.1)
GO.CC.HS.Flower <- analyze.all.GO(idsHS.Flower, aspect="CC", only.FDR.le=0.1)

GO.BP.HS.Growing <- analyze.all.GO(idsHS.Growing, aspect="BP", only.FDR.le=0.1)
GO.MF.HS.Growing <- analyze.all.GO(idsHS.Growing, aspect="MF", only.FDR.le=0.1)
GO.CC.HS.Growing <- analyze.all.GO(idsHS.Growing, aspect="CC", only.FDR.le=0.1)

GO.BP.HS.Maturity <- analyze.all.GO(idsHS.Maturity, aspect="BP", only.FDR.le=0.1)
GO.MF.HS.Maturity <- analyze.all.GO(idsHS.Maturity, aspect="MF", only.FDR.le=0.1)
GO.CC.HS.Maturity <- analyze.all.GO(idsHS.Maturity, aspect="CC", only.FDR.le=0.1)

# Summarizing, Flower, Growing, Maturity.
# Will analyze only odds>2 (discarding all others and filtering)
# Flower
GO.BP.HS.Flower <- GO.BP.HS.Flower[order(GO.BP.HS.Flower$odds, decreasing=T),]
GO.CC.HS.Flower <- GO.CC.HS.Flower[order(GO.CC.HS.Flower$odds, decreasing=T),]
GO.MF.HS.Flower <- GO.MF.HS.Flower[order(GO.MF.HS.Flower$odds, decreasing=T),]
GO.BP.HS.Flower <- GO.BP.HS.Flower[GO.BP.HS.Flower$odds >2,]
GO.CC.HS.Flower <- GO.CC.HS.Flower[GO.CC.HS.Flower$odds >2,]
GO.MF.HS.Flower <- GO.MF.HS.Flower[GO.MF.HS.Flower$odds >2,]
GO.HS.Flower <- rbind(GO.BP.HS.Flower, GO.MF.HS.Flower, GO.CC.HS.Flower)

# Growing
GO.BP.HS.Growing <- GO.BP.HS.Growing[order(GO.BP.HS.Growing$odds, decreasing=T),]
GO.CC.HS.Growing <- GO.CC.HS.Growing[order(GO.CC.HS.Growing$odds, decreasing=T),]
GO.MF.HS.Growing <- GO.MF.HS.Growing[order(GO.MF.HS.Growing$odds, decreasing=T),]
GO.BP.HS.Growing <- GO.BP.HS.Growing[GO.BP.HS.Growing$odds >2,]
GO.CC.HS.Growing <- GO.CC.HS.Growing[GO.CC.HS.Growing$odds >2,]
GO.MF.HS.Growing <- GO.MF.HS.Growing[GO.MF.HS.Growing$odds >2,]
GO.HS.Growing <- rbind(GO.BP.HS.Growing, GO.MF.HS.Growing, GO.CC.HS.Growing)

# Maturity
GO.BP.HS.Maturity <- GO.BP.HS.Maturity[order(GO.BP.HS.Maturity$odds, decreasing=T),]
GO.CC.HS.Maturity <- GO.CC.HS.Maturity[order(GO.CC.HS.Maturity$odds, decreasing=T),]
GO.MF.HS.Maturity <- GO.MF.HS.Maturity[order(GO.MF.HS.Maturity$odds, decreasing=T),]
GO.BP.HS.Maturity <- GO.BP.HS.Maturity[GO.BP.HS.Maturity$odds >2,]
GO.CC.HS.Maturity <- GO.CC.HS.Maturity[GO.CC.HS.Maturity$odds >2,]
GO.MF.HS.Maturity <- GO.MF.HS.Maturity[GO.MF.HS.Maturity$odds >2,]
GO.HS.Maturity <- rbind(GO.BP.HS.Maturity, GO.MF.HS.Maturity, GO.CC.HS.Maturity)

# Those data frames were edited and ordered to produce the tables presented.
-------------------------------------------------------------------------------------
```

All objects produced as results of the R computations are available upon request in the binary R object "`ObjectsForSupplementary.RData`".
